# Archaeal “Dark Matter” and the Origin of Eukaryotes

**DOI:** 10.1093/gbe/evu031

**Published:** 2014-02-14

**Authors:** Tom A. Williams, T. Martin Embley

**Affiliations:** Institute for Cell and Molecular Biosciences, Newcastle University, Newcastle upon Tyne, United Kingdom

**Keywords:** eukaryogenesis, phylogenetics, “dark matter”, Tree of Life

## Abstract

Current hypotheses about the history of cellular life are mainly based on analyses of cultivated organisms, but these represent only a small fraction of extant biodiversity. The sequencing of new environmental lineages therefore provides an opportunity to test, revise, or reject existing ideas about the tree of life and the origin of eukaryotes. According to the textbook three domains hypothesis, the eukaryotes emerge as the sister group to a monophyletic Archaea. However, recent analyses incorporating better phylogenetic models and an improved sampling of the archaeal domain have generally supported the competing eocyte hypothesis, in which core genes of eukaryotic cells originated from within the Archaea, with important implications for eukaryogenesis. Given this trend, it was surprising that a recent analysis incorporating new genomes from uncultivated Archaea recovered a strongly supported three domains tree. Here, we show that this result was due in part to the use of a poorly fitting phylogenetic model and also to the inclusion by an automated pipeline of genes of putative bacterial origin rather than nucleocytosolic versions for some of the eukaryotes analyzed. When these issues were resolved, analyses including the new archaeal lineages placed core eukaryotic genes within the Archaea. These results are consistent with a number of recent studies in which improved archaeal sampling and better phylogenetic models agree in supporting the eocyte tree over the three domains hypothesis.

## Introduction

Current estimates suggest that sequenced genomes represent only a tiny fraction of extant microbial diversity, and that much of the microbial world remains unknown (Rappe and Giovannoni 2003). Exploration of this microbial “dark matter” (Marcy et al. 2007) holds tremendous potential for improving our understanding of the diversity and evolution of life on Earth. Among prokaryotic groups, the Archaea are particularly poorly sampled but, in addition to their environmental abundance and importance in the global cycling of carbon and nitrogen (Pester et al. 2011), they are crucially important for understanding the origin of eukaryotes. In the traditional three domains tree, the host cell for the mitochondrial endosymbiont was part of a third domain of cellular life that split from the Archaea before the diversification of either group (Woese et al. 1990). The main alternative to this view is that the host cell was a fully fledged Archaeon, implying that eukaryotes originated in a partnership between a bacterial endosymbiont and an archaeal host cell (Lake et al. 1984; Martin and Muller 1998; reviewed in Williams et al. 2013); this view has gained increased support from phylogenies that place core eukaryotic genes, including ribosomal RNA and proteins, within the Archaea (Cox et al. 2008; Foster et al. 2009; Kelly et al. 2011; Williams et al. 2012; Lasek-Nesselquist and Gogarten 2013). In particular, recent phylogenies have placed these core eukaryotic genes within, or as the sister group to, the TACK superphylum of Archaea (Guy and Ettema 2011) comprising the Thaumarchaeota (Brochier-Armanet et al. 2008), Aigarchaeota (Nunoura et al. 2011), Crenarchaeota (or eocytes), and Korarchaeota (Elkins et al. 2008), consistent with an extended version of the eocyte hypothesis of Lake et al. (1984).

The recent publication of the most comprehensive survey of uncultured microbial diversity to date (Rinke et al. 2013) has provided an unprecedented wealth of valuable new genomic data to refine the phylogenetic position of core eukaryotic genes and to test hypotheses for eukaryotic origins. Genomes from new archaeal lineages are particularly welcome because improvements in taxon sampling are generally expected to increase the reliability of the resulting phylogenetic trees (Graybeal 1998). Interestingly, an initial phylogenetic analysis of 38 protein-coding genes shared between Bacteria, eukaryotes, and an expanded sampling of Archaea from the Genomic Encyclopedia of Bacteria and Archaea (GEBA) project recovered a strongly supported three domains tree in which eukaryotes branched outside a monophyletic Archaea (Rinke et al. 2013). This result was particularly striking because previous improvements in archaeal sampling, including the sequencing of organisms from the TACK superphylum (Guy and Ettema 2011), have otherwise favored topologies consistent with archaeal-host hypotheses rather than the traditional three domains tree (Lasek-Nesselquist and Gogarten 2013; Williams et al. 2012). In this study, we have investigated the possible reasons for the disagreement between these previous studies and the recent analyses of Rinke et al. (2013).

## Materials and Methods

### Sequences and Alignments

The sequence alignments and tree files generated as part of these analyses have been deposited on Figshare (http://figshare.com/articles/Supplementary_data_files_for_Archaeal_dark_matter_and_the_origin_of_eukaryotes_/926485; DOI: http://www.dx.doi.org/10.6084/m9.figshare.926485, last accessed February 23, 2014). Single gene trees for the individual genes of the original Rinke et al. (2013) concatenation were built using RAxML 7.7.2 (Stamatakis 2006) with the LG + F substitution model and 200 rapid bootstraps. Putative mitochondrial and plastid genes were identified as eukaryotic sequences grouping with, or within, the Bacteria with strong support (≥70% bootstrap support) in single-gene phylogenies. For each of the cases so identified, we confirmed that they were annotated as mitochondrial or plastid sequences in NCBI GenBank. In the case of triose phosphate isomerase, published analyses support its secondary acquisition by eukaryotes from Bacteria (Keeling and Doolittle 1997). Full details of each of the organellar genes identified in this way are provided in supplementary table S1, Supplementary Material online. In updating the original concatenation, we replaced the detected organellar sequences with their nucleocytoplasmic homologs (i.e., the orthologs of the other eukaryotic sequences in the alignment), where possible. We then built new single-gene trees to confirm that the appropriate replacement sequences had been found, by confirming the monophyly of the eukaryotic clade. The genes were aligned, and the alignments edited, as described in Rinke et al. (2013).

We assigned orthologs from the newly sequenced archaeal genomes to our existing 29-gene data set using Cognitor (Tatusov et al. 2003). The protein sequences were aligned using Muscle (Edgar 2004), Mafft (Katoh et al. 2005), ProbCons (Do et al. 2005), Kalign (Lassmann and Sonnhammer 2005), and Fsa (Bradley et al. 2009), and a consensus alignment generated with T-Coffee (Notredame et al. 2000). Poorly aligning positions were detected and removed with BMGE (Criscuolo and Gribaldo 2010) using the BLOSUM30 matrix to score conservation.

### Phylogenetics

The analyses with single-matrix models used amino acid frequencies inferred from the data by maximum likelihood; both single-matrix and site-heterogeneous analyses employed a discrete approximation to the gamma distribution with four rate categories (Yang 1994) for modeling across-site rate variation. The best fitting single-matrix substitution models were chosen using ProtTest3 (Darriba et al. 2011). To compare the fit of single-matrix and site-heterogeneous models in a Bayesian context, we used posterior predictive simulations (Bollback 2002) as implemented in the ppred program of the PhyloBayes package (http://www.phylobayes.org, last accessed February 23, 2014). Maximum likelihood phylogenies were inferred using RAxML 7.7.2, using 200 rapid bootstraps for each tree. Bayesian analyses were performed using PhyloBayes 3.3 (Lartillot et al. 2009) and PhyloBayes MPI 1.5a (Lartillot et al. 2013). We ran two independent MCMC chains for each analysis, and used the included bpcomp and tracecomp programs to generate convergence diagnostics. Chains were stopped when the maximum discrepancy in bipartition frequencies and several additional summary variables (including the alpha parameter for across-site rate variation, tree length, mean posterior log-likelihood) between the two chains dropped below 0.1, and the effective sizes of the summary variables were all more than 100, as recommended by the authors.

## Results and Discussion

### Analysis of the Original Dark Matter Supermatrix

**Fig. 1.—:**
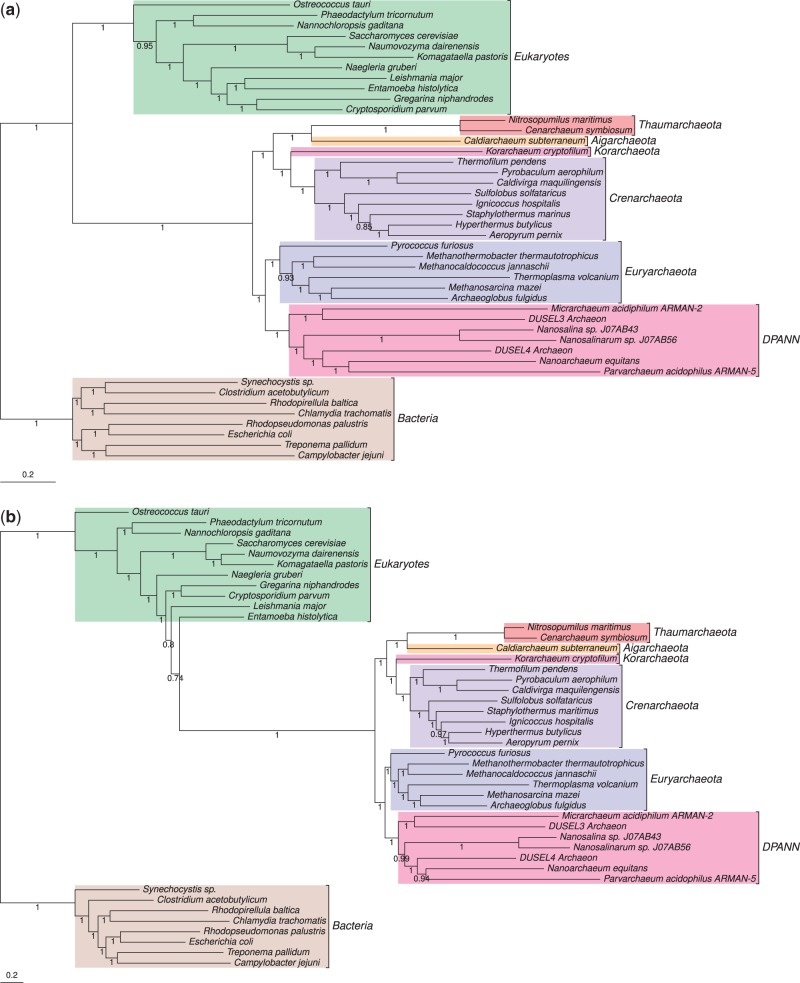
Bayesian phylogenies inferred from the dark matter supermatrix of Rinke et al. (2013). (*a*) The consensus tree inferred under the best-fitting LG single matrix model. This is a three domains (Woese et al. 1990) tree, with maximal support (PP = 1) for archaeal monophyly. (*b*) The tree inferred under the CAT + GTR model for this data set does not correspond to any published hypothesis on the tree of life, with the Archaea emerging from within a paraphyletic eukaryotic clade; this topology is likely due to contamination of the eukaryotic data set with genes of mitochondrial and plastid origin. Our interpretation is based on a root for the tree of life within the Bacteria (Cavalier-Smith 2006; Lake et al. 2009), or on the bacterial stem (Gogarten et al. 1989; Iwabe et al. 1989; Dagan et al. 2010). Branch lengths are proportional to expected numbers of substitutions per site, and support values are Bayesian posterior probabilities.

The initial dark matter phylogeny providing support for the three domains tree was inferred under the Jones–Taylor–Thornton (JTT) phylogenetic model (Jones et al. 1992) from a concatenation (supermatrix; de Queiroz and Gatesy 2007) of 38 protein-coding genes. As the fit of the phylogenetic model to the data has previously been shown to play an important role in the recovery of a three domains or eocyte tree (Cox et al. 2008; Foster et al. 2009; Lasek-Nesselquist and Gogarten 2013; Williams et al. 2013), we first investigated the fit of this model to the original dark matter protein supermatrix. Model selection using ProtTest3 (Darriba et al. 2011) suggested that the alternative single matrix LG model (Le and Gascuel 2008) provided a better fit to the data under both the Akaike Information Criterion and the Bayesian Information Criterion than the JTT model. A Bayesian phylogenetic analysis using the LG model (fig. 1*a*) recovered a three domains tree with maximal support (posterior probability of 1 for archaeal monophyly). However, even the best-fitting single-matrix model (in this case, LG) may provide a relatively poor fit to data sets containing highly divergent sequences (Quang le et al. 2008; Williams et al. 2012). In particular, single-matrix models do not account for variation in sequence composition across sites, which may lead to tree reconstruction artifacts such as long-branch attraction (LBA; Philippe et al. 2011). We therefore investigated whether the more flexible CAT + GTR site mixture model (Lartillot and Philippe 2004) also favored the three domains over the eocyte hypothesis for this data set. Posterior predictive tests (Bollback 2002) suggested that the CAT + GTR model fits the data better than the LG model, at least with respect to modeling the site-specific biochemical properties of the alignment (*P* = 0.057 for CAT + GTR, *P* = 0 for LG). This feature of sequence data is considered particularly important because accurate modeling of site-specific selective constraints helps to distinguish molecular homoplasies (convergent evolution) from synapomorphies (historical signal), potentially mitigating the effects of LBA (Lartillot et al. 2007). Surprisingly, the tree inferred under the best fitting CAT + GTR model did not support either the three domains or eocyte hypotheses, or indeed any other established hypothesis for the tree of life (fig. 1*b*); instead it supports an unexpected scenario in which core archaeal genes were derived from within the eukaryotic radiation (fig. 1*b*).

To investigate the origin of the apparent signal for eukaryotic paraphyly in the dark matter supermatrix, we built trees for each of the 38 genes included in the original concatenation (Rinke et al. 2013). The results of this analysis were surprising: for 18 of the 38 genes, the eukaryotes were not monophyletic because one or more of the eukaryotic sequences clustered within the Bacteria (supplementary fig. S1 [Supplementary Material online] and summarized in supplementary table S1 [Supplementary Material online]). In these 18 trees, the other eukaryotes formed a clade either within the Archaea (16/18), as their sister group (1/18), or were interspersed with archaeal homologs (1/18). For 12 additional genes eukaryotes grouped within the Bacteria (8/12), comprised only two eukaryotic sequences (2/12), or the genes have apparently been lost from eukaryotes and/or Archaea (2/12). The observed nonmonophyly of eukaryotes in the 18 single gene trees can be explained in part by the inclusion of mitochondrial and plastid sequences in the eukaryotic data set (supplementary fig. S1, Supplementary Material online). For example, all of the bacterial-like *Saccharomyces cerevisiae* sequences are annotated in the NCBI RefSeq database as mitochondrial genes (supplementary table S2, Supplementary Material online). The phenyl-tRNA ligase of *Phaeodactylum tricornutum* groups strongly with the cyanobacterium *Synechocystis*, consistent with a plastid origin (supplementary fig. S1, Supplementary Material online). These organellar sequences are not useful for testing the three domains/eocyte question because they trace their ancestry to the free-living ancestors of the mitochondrion or plastid rather than to the eukaryotic host cell lineage. Moreover, the inclusion of mitochondrial and plastid sequences in the eukaryotic data is expected to weight the analysis against the eocyte topology, because it will tend to draw the eukaryotes and Bacteria together in the tree—as can be seen most clearly in figure 1*b*.

**Fig. 2.—:**
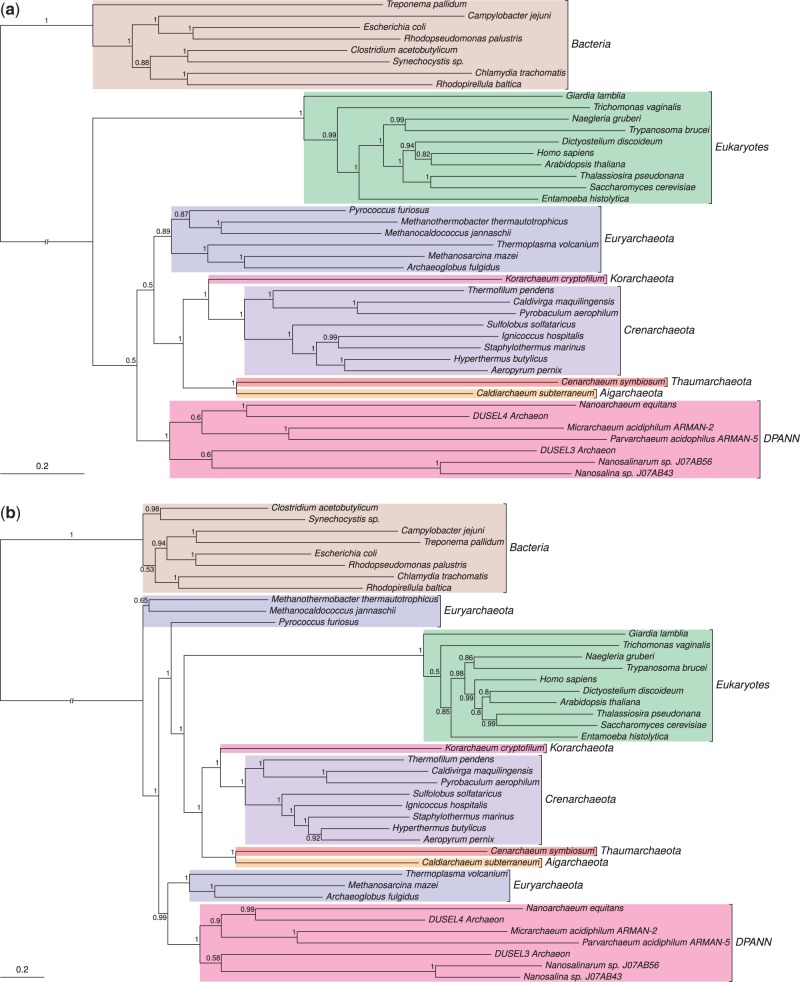
Bayesian phylogenies inferred from the dark matter data set after eukaryotic genes of bacterial origin had been replaced with their nucleocytosolic homologues. (*a*) Inference under the LG model recovers a weakly supported three domains tree, with support for archaeal monophyly reduced to 0.5. (*b*) The better-fitting CAT + GTR model recovers a strongly supported eocyte tree, with core eukaryotic genes forming a clade with the TACK superphylum of Archaea with maximum support (PP = 1). Our interpretation is based on a root for the tree of life within the Bacteria (Cavalier-Smith 2006; Lake et al. 2009), or on the bacterial stem (Gogarten et al. 1989; Iwabe et al. 1989; Dagan et al. 2010). Branch lengths are proportional to expected numbers of substitutions per site, and support values are Bayesian posterior probabilities.

To investigate further, the automatic gene selection, alignment, and masking pipeline (Darling et al. 2014) that was used in Rinke et al. (2013) was rerun, but additional checks for excluding eukaryotic genes of putative mitochondrial and plastid origin, and for improving taxonomic representation, were included. The new alignment produced by the automatic pipeline contained 20 genes. Eighteen genes from the original data set were removed because these genes had only a patchy distribution in Archaea and/or eukaryotes (supplementary table S3, Supplementary Material online) as determined by the PhyloSift pipeline (Darling et al. 2014). As before, we used ProtTest3 (Darriba et al. 2011) to select the best-fitting single-matrix model (LG) for the new 20-gene alignment, and also evaluated the fit of the more flexible CAT + GTR model. Posterior predictive simulations (Bollback 2002) indicated that CAT + GTR, but not LG, adequately accounted for the site-specific biochemical properties of the alignment (*P* = 0.069 for CAT + GTR, *P* = 0 for LG) (Lartillot et al. 2007). For this supermatrix, we inferred a weakly supported three domains tree under the LG model, with a posterior probability of 0.5 for archaeal monophyly (fig. 2*a*). By contrast, the better-fitting CAT + GTR model recovered a maximally supported eocyte tree (fig. 2*b*). The results from this 20-gene data set are therefore consistent with previous analyses in which improving the fit of the phylogenetic model weakened support for the three domains hypothesis and led to the recovery of an eocyte tree (Cox et al. 2008; Foster et al. 2009; Lasek-Nesselquist and Gogarten 2013). The mitochondrial and plastid contamination of the original data set appears to have been an important factor in these results, as can be seen by comparing the trees inferred under the best-fitting model (CAT + GTR) before and after these sequences were removed (figs. 1*b* and 2*b*).

### A Complementary Data Set for Investigating Eukaryotic Host Cell Origins

Phylogenetic analyses aimed at understanding the origin of the eukaryotic host cell have typically focused on a broadly conserved core of 30–40 genes that are primarily involved in translation, and which appear to be more resistant to lineage-specific loss and horizontal transfer than other genes (Rivera et al. 1998). Published analyses of these genes have used overlapping subsets of this conserved core due to differences in taxonomic sampling and the protocols used to select phylogenetic markers. In a previous analysis of the relationship between eukaryotic and archaeal core genes (Williams et al. 2012), we used a set of 29 single-copy orthologs conserved in a representative taxonomic sample of Archaea, Eukaryotes, and Bacteria. The overlap (16 genes) between that data set and the 38 genes originally used by Rinke et al. (2013) is modest (supplementary table S3, Supplementary Material online). This is due in part to the different starting points for our ortholog searches—Bacteria for Rinke et al. (2013), and the eukaryotic red alga *Cyanidioschyzon merolae* in our previous work (Cox et al. 2008; Williams et al. 2012). Another factor in the differences between the two data sets was the requirement by Williams et al. (2012) that the selected genes be conserved as single-copy orthologs across all ten eukaryotic genomes analyzed. The representation of eukaryotes in the automatically generated data set of Rinke et al. (2013) was more variable: of 11 eukaryotic genomes included in the analysis, a mean of 7.8 (range 0–11) were represented in each single gene alignment.

**Fig. 3.—:**
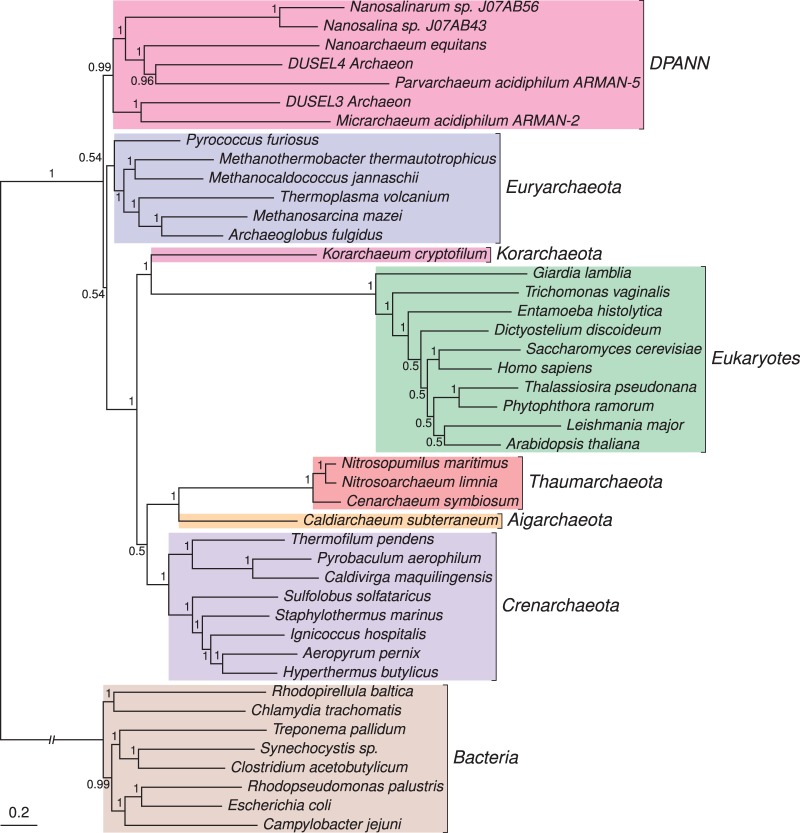
Bayesian concatenated protein phylogeny inferred from a congruent set of 29 genes conserved in Bacteria, Archaea, and eukaryotes. The eukaryotes emerge from within the TACK superphylum of Archaea with maximal support. There is strong support (PP = 0.99) for the monophyly of *Nanoarchaeum equitans* with the newly sequenced “DPANN” Archaea. These are the 29 genes from Williams et al. (2012), updated to include the new archaeal sequences from the GEBA project (Rinke et al. 2013). The tree was inferred using the CAT + GTR model in PhyloBayes MPI (Lartillot et al. 2013). Our interpretation is based on a root for the tree of life within the Bacteria (Cavalier-Smith 2006; Lake et al. 2009), or on the bacterial stem (Gogarten et al. 1989; Iwabe et al. 1989; Dagan et al. 2010). Branch lengths are proportional to expected numbers of substitutions per site, and support values are Bayesian posterior probabilities.

We updated the Williams et al. (2012) 29-gene data set with orthologs from the newly sequenced archaeal genomes using Cognitor (Tatusov et al. 2003), and inferred a Bayesian phylogeny using the CAT + GTR model from the concatenated alignment (fig. 3). This analysis agreed with the CAT + GTR tree inferred from the new 20 gene version of the Rinke et al. (2013) data set in placing the eukaryotes within the Archaea as the closest relatives of the TACK superphylum, and recovering a clade containing *Nanoarchaeum*, the Nanohaloarchaeota (*Nanosalinarum* and *Nanosalina* sp.; Narasingarao et al. 2012), the ARMAN lineages (Baker et al. 2006), and the new DPANN Archaea with strong support (PP = 0.99). It may be that the improved sampling achieved by Rinke et al. (2013) has helped to stabilize the position of these previously problematic taxa (Brochier-Armanet et al. 2011) in phylogenetic trees. Our analyses also suggest that the position of the DPANN clade as a whole within the Archaea is still somewhat ambiguous, although they are excluded from the TACK/eukaryote clade in all of our analyses. The analysis also recovered *Korarchaeum* as the closest relative of the eukaryotes, a result also obtained previously (Williams et al. 2012). The recovery of an eocyte tree, rather than the three domains tree, from both data sets suggests that this result is robust to the choice of genes, alignment methods, or masking protocols.

## Conclusions

The Genomic Encyclopaedia of microbial dark matter (Rinke et al. 2013) represents a tremendous scientific and technical achievement with the potential to dramatically improve our understanding of the natural microbial world. The project has already provided new insights into the metabolic diversity of prokaryotes, and the wealth of new genome data is likely to stimulate much future work on microbial evolution and ecology. Here, we have investigated the impact of the newly sequenced archaeal lineages on support for the three domains and eocyte trees. Deciding which of these trees is better supported in the light of the new data is important because they underpin contrasting hypotheses for the origin of eukaryotic cells and the host for the mitochondrial endosymbiont (Williams et al. 2013). In the original dark matter paper, it was suggested that the new data were not consistent with the eocyte hypothesis, and indeed a strongly supported three domains tree was recovered in those initial analyses (Rinke et al. 2013). This result was surprising because prior improvements in archaeal sampling had tended to weaken, rather than strengthen, support for the three domains tree (Guy and Ettema 2011; Kelly et al. 2011; Williams et al. 2012, 2013; Lasek-Nesselquist and Gogarten 2013). Here, we demonstrate that the preference for the three domains tree was driven in part by the inclusion of genes of bacterial origin for eukaryotes in the original, automatically generated dark matter alignments. When this issue was addressed in a broadly sampled subset of the original supermatrix, a weakly supported three domains tree was inferred under the single-matrix LG model, but a strongly supported eocyte tree was inferred under the better fitting CAT + GTR model (fig. 2). Addition of the new archaeal sequences to a previously published data set (Williams et al. 2012) also provided strong support for an eocyte topology using the CAT + GTR model. These results, incorporating the newly discovered archaeal dark matter, are thus in line with recent analyses that converge on a version of the eocyte hypothesis in which core eukaryotic genes are related to those of the TACK Archaea, rather than the alternative three domains tree (Williams et al. 2013).

## Supplementary Material

Supplementary figures S1 and tables S1–S3 are available at *Genome Biology and Evolution* online (http://www.gbe.oxfordjournals.org/).

Supplementary Data
