# Supplementary material for: Archaeal “Dark Matter” and the Origin of Eukaryotes
Source: Genome Biol Evol. 2014 Feb 14;6(3):474–81. doi: 10.1093/gbe/evu031 (PMC3971582; doi:10.1093/gbe/evu031)
Supplement: Supplementary Data [file supp_6_3_474__index.html]

Archaeal “dark matter” and the origin of eukaryotes — Archaeal “Dark Matter” and the Origin of Eukaryotes — Supplementary Data 

# Archaeal “Dark Matter” and the Origin of Eukaryotes

## Supplementary Data

files

**Files in this Data Supplement:**

- Supplementary Data - pdf file
- Supplementary Data - docx file
