# Supplementary material for: Archaeal “Dark Matter” and the Origin of Eukaryotes
Source: Genome Biol Evol. 2014 Feb 14;6(3):474–81. doi: 10.1093/gbe/evu031 (PMC3971582; doi:10.1093/gbe/evu031)
Supplement: Supplementary Data [file supp_evu031_Figure_S1.pdf]

Supplementary Figure 1: Single gene trees for the 38 genes included in the concatenated alignment of Rinke et al. (2013)

Methods

Single gene trees were inferred using the LG+F model in RAxML 7.7.2 with 200 bootstrap replicates. The alignments used are the same as those of Rinke et al. (2013). Trees were sorted into the following categories:

- Those containing mitochondrial or plastid contamination (that is, where one or more eukaryotes cluster within the Bacteria);
- Those free of contamination (and suitable for phylogenetic analysis);
- Bacterial genes, or genes for which a nucleo-cytosolic orthologue does not exist (triose phosphate isomerase and the mitochondrial gene Guf1);
- Non-conserved genes, which are not found in one or more domains, or for which one two eukaryotic homologues were available.

Key

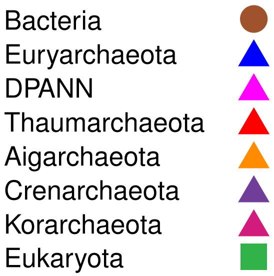

Genes containing mitochondrial or plastid contamination

Rpl23bp

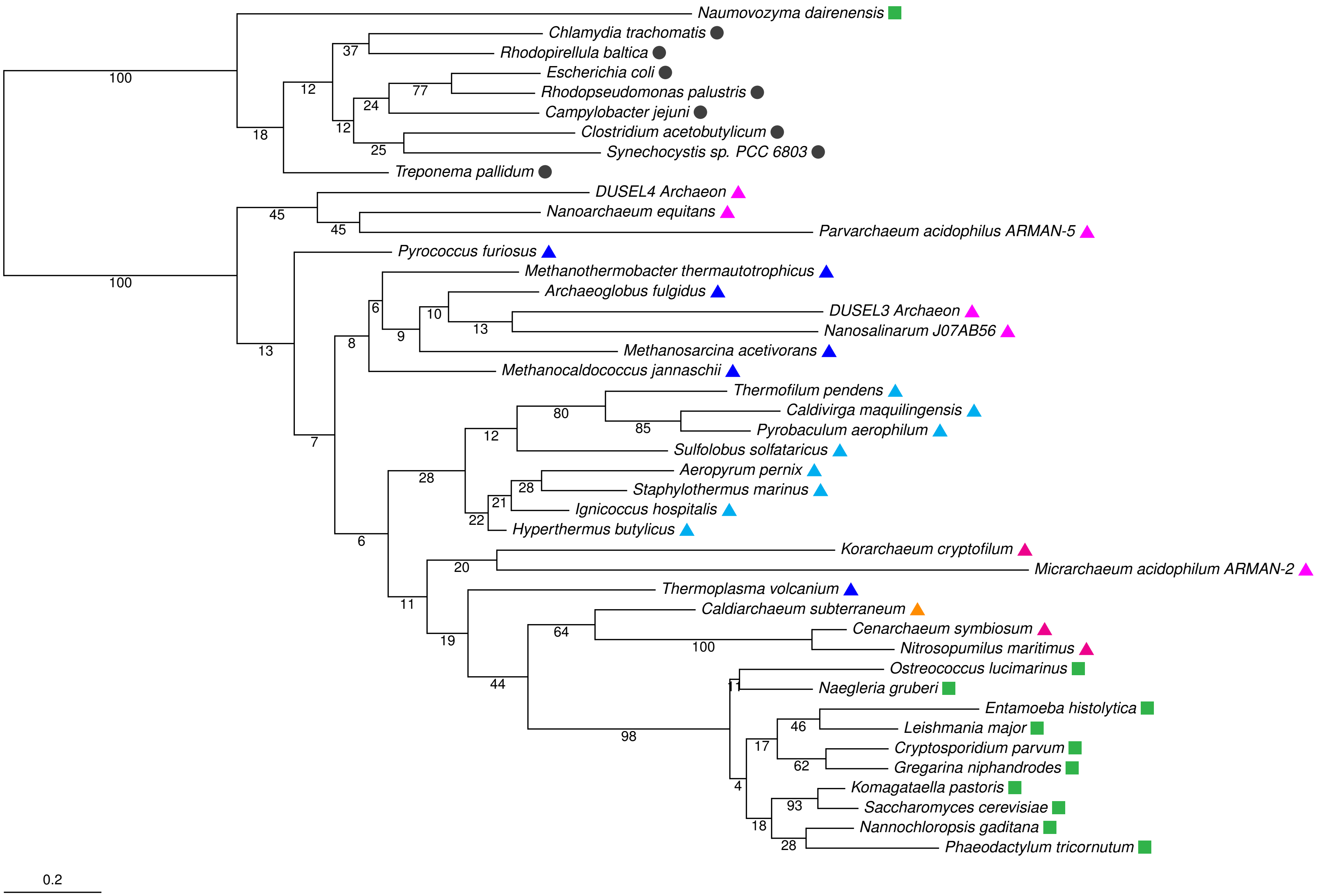

Kae1p

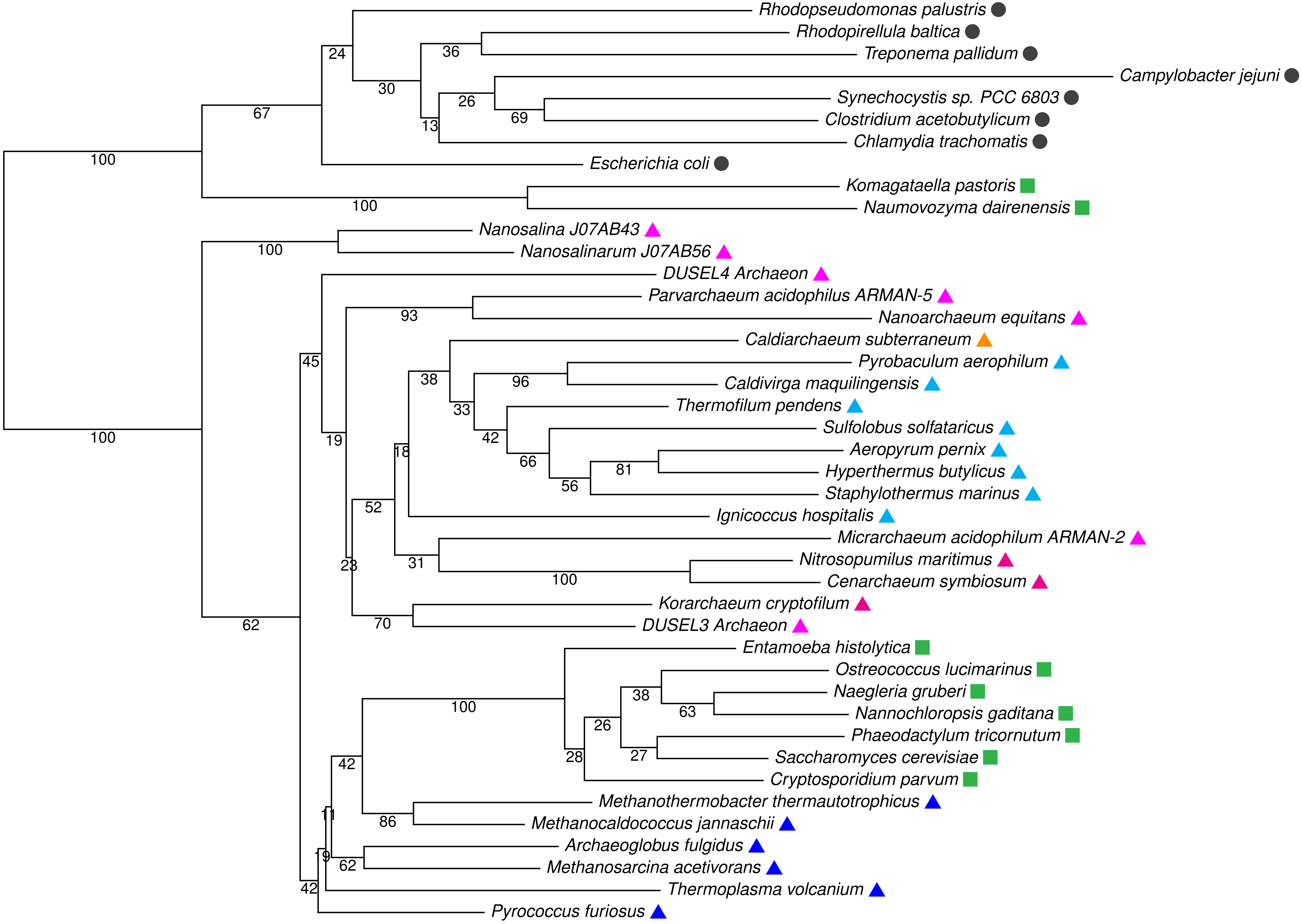
Rhodopseudomonas palustris
Rhodopirellula baltica
Treponema pallidum
Campylobacter jejuni
Synechocystis sp. PCC 6803
Clostridium acetobutylicum
Chlamydia trachomatis
Escherichia coli
Komagataella pastoris
Naumovozyma dairenensis
Nanosalina J07AB43
Nanosalinarum J07AB56
DUSEL4 Archaeon
Parvarchaeum acidophilus ARMAN-5
Nanoarchaeum equitans
Caldiarchaeum subterraneum
Pyrobaculum aerophilum
Caldivirga maquilingensis
Thermofilum pendens
Sulfolobus solfataricus
Aeropyrum pernix
Hyperthermus butylicus
Staphylothermus marinus
Ignicoccus hospitalis
Micrarchaeum acidophilum ARMAN-2
Nitrosopumilus maritimus
Cenarchaeum symbiosum
Korarchaeum cryptofilum
DUSEL3 Archaeon
Entamoeba histolytica
Ostreococcus lucimarinus
Naegleria gruberi
Nannochloropsis gaditana
Phaeodactylum tricornutum
Saccharomyces cerevisiae
Cryptosporidium parvum
Methanothermobacter thermautotrophicus
Methanocaldococcus jannaschii
Archaeoglobus fulgidus
Methanosarcina acetivorans
Thermoplasma volcanium
Pyrococcus furiosus
24
36
30
26
69
13
67
100
100
100
100
93
45
38
96
33
42
19
18
66
81
56
52
31
100
23
70
62
100
38
63
26
27
28
42
11
86
19
62
42

Rps2p

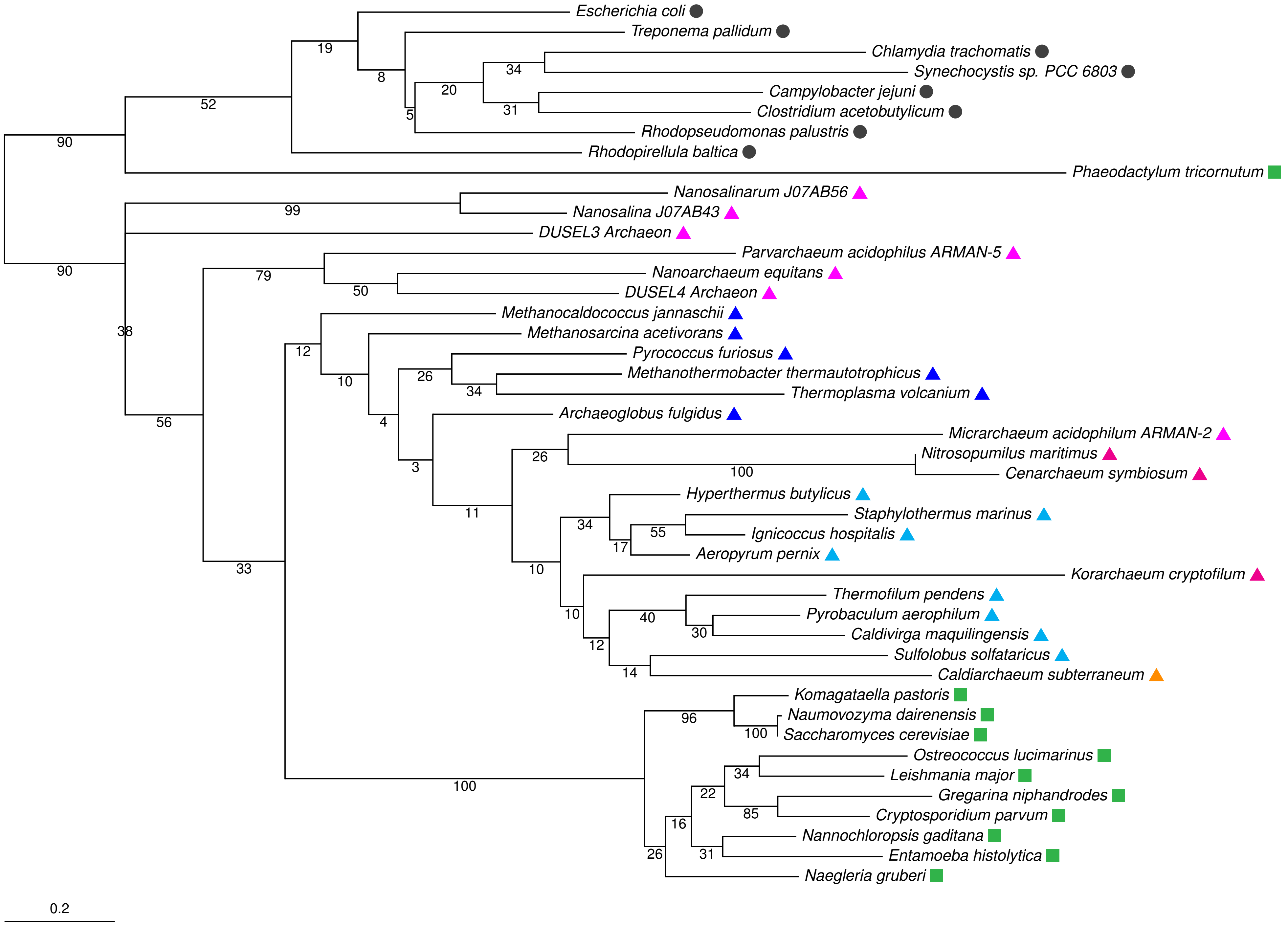

Escherichia coli
Treponema pallidum
Chlamydia trachomatis
Synechocystis sp. PCC 6803
Campylobacter jejuni
Clostridium acetobutylicum
Rhodopseudomonas palustris
Rhodopirellula baltica
Phaeodactylum tricornutum
Nanosalinarum J07AB56
Nanosalina J07AB43
DUSEL3 Archaeon
Parvarchaeum acidophilus ARMAN-5
Nanoarchaeum equitans
DUSEL4 Archaeon
Methanocaldococcus jannaschii
Methanosarcina acetivorans
Pyrococcus furiosus
Methanothermobacter thermautotrophicus
Thermoplasma volcanium
Archaeoglobus fulgidus
Micrarchaeum acidophilum ARMAN-2
Nitrosopumilus maritimus
Cenarchaeum symbiosum
Hyperthermus butylicus
Staphylothermus marinus
Ignicoccus hospitalis
Aeropyrum pernix
Korarchaeum cryptofilum
Thermofilum pendens
Pyrobaculum aerophilum
Caldivirga maquilingensis
Sulfolobus solfataricus
Caldiarchaeum subterraneum
Komagataella pastoris
Naumovozyma dairenensis
Saccharomyces cerevisiae
Ostreococcus lucimarinus
Leishmania major
Gregarina niphandrodes
Cryptosporidium parvum
Nannochloropsis gaditana
Entamoeba histolytica
Naegleria gruberi
0.2

Rpl9p

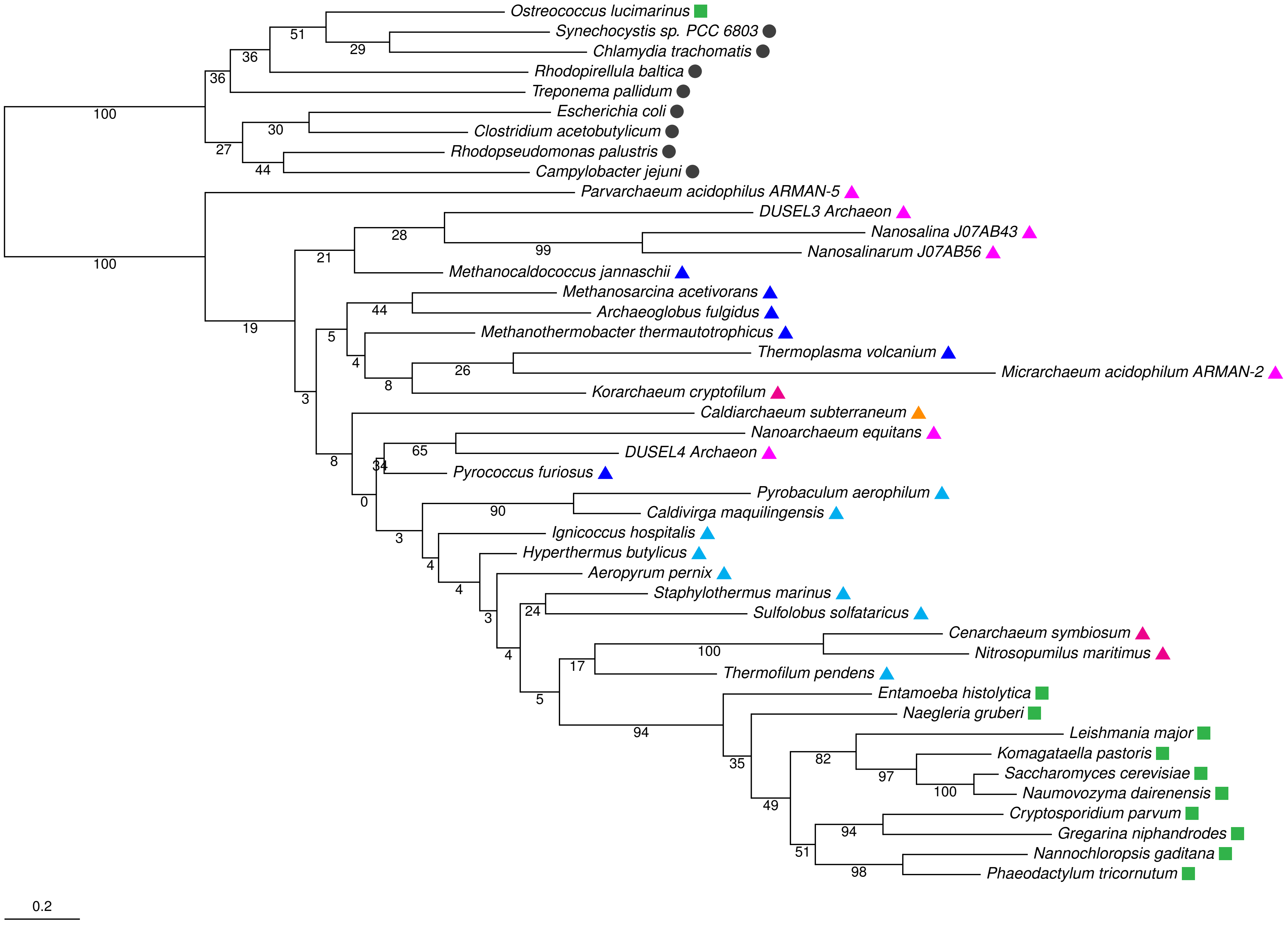
Ostreococcus lucimarinus
Synechocystis sp. PCC 6803
Chlamydia trachomatis
Rhodopirellula baltica
Treponema pallidum
Escherichia coli
Clostridium acetobutylicum
Rhodopseudomonas palustris
Campylobacter jejuni
Parvarchaeum acidophilus ARMAN-5
DUSEL3 Archaeon
Nanosalina J07AB43
Nanosalinarum J07AB56
Methanocaldococcus jannaschii
Methanosarcina acetivorans
Archaeoglobus fulgidus
Methanothermobacter thermautotrophicus
Thermoplasma volcanium
Micrarchaeum acidophilum ARMAN-2
Korarchaeum cryptofilum
Caldiarchaeum subterraneum
Nanoarchaeum equitans
DUSEL4 Archaeon
Pyrococcus furiosus
Pyrobaculum aerophilum
Caldivirga maquilingensis
Ignicoccus hospitalis
Hyperthermus butylicus
Aeropyrum pernix
Staphylothermus marinus
Sulfolobus solfataricus
Cenarchaeum symbiosum
Nitrosopumilus maritimus
Thermofilum pendens
Entamoeba histolytica
Naegleria gruberi
Leishmania major
Komagataella pastoris
Saccharomyces cerevisiae
Naumovozyma dairenensis
Cryptosporidium parvum
Gregarina niphandrodes
Nannochloropsis gaditana
Phaeodactylum tricornutum
0.2

Rpl17ap

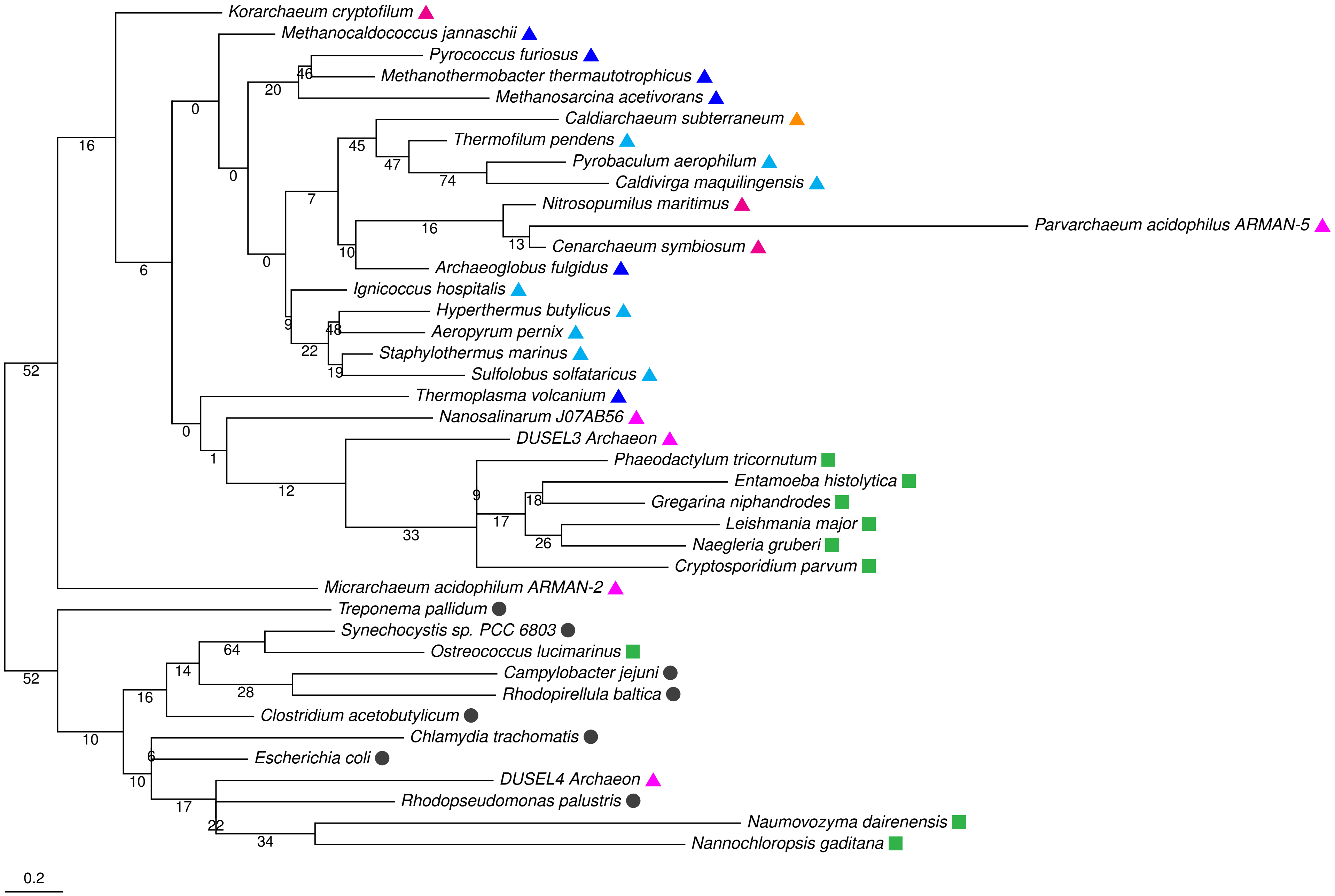

Korarchaeum cryptofilum
Methanocaldococcus jannaschii
Pyrococcus furiosus
Methanothermobacter thermautotrophicus
Methanosarcina acetivorans
Caldiarchaeum subterraneum
Thermofilum pendens
Pyrobaculum aerophilum
Caldivirga maquilingensis
Nitrosopumilus maritimus
Parvarchaeum acidophilus ARMAN-5
Cenarchaeum symbiosum
Archaeoglobus fulgidus
Ignicoccus hospitalis
Hyperthermus butylicus
Aeropyrum pernix
Staphylothermus marinus
Sulfolobus solfataricus
Thermoplasma volcanium
Nanosalinarum J07AB56
DUSEL3 Archaeon
Phaeodactylum tricornutum
Entamoeba histolytica
Gregarina niphandrodes
Leishmania major
Naegleria gruberi
Cryptosporidium parvum
Micrarchaeum acidophilum ARMAN-2
Treponema pallidum
Synechocystis sp. PCC 6803
Ostreococcus lucimarinus
Campylobacter jejuni
Rhodopirellula baltica
Clostridium acetobutylicum
Chlamydia trachomatis
Escherichia coli
DUSEL4 Archaeon
Rhodopseudomonas palustris
Naumovozyma dairenensis
Nannochloropsis gaditana
0.2

Mrp9p

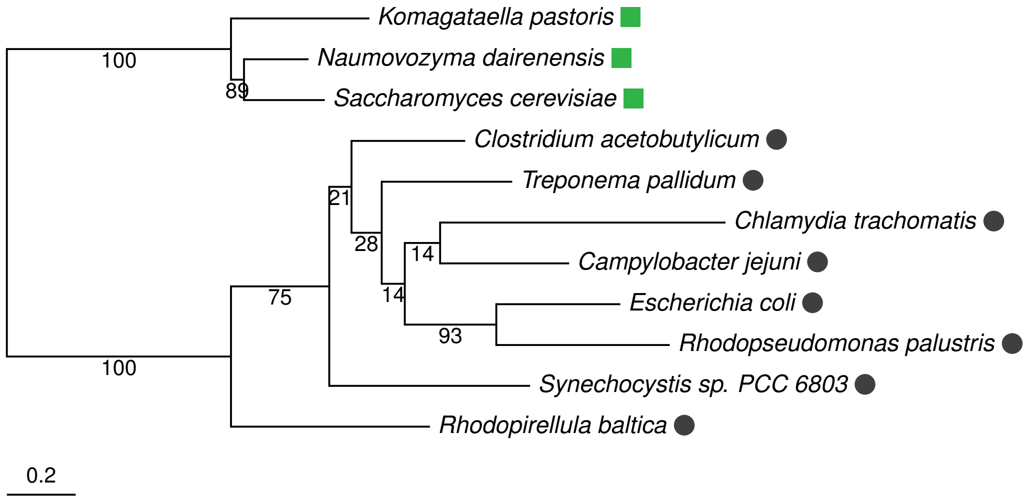
Komagataella pastoris
Naumovozyma dairenensis
Saccharomyces cerevisiae
Clostridium acetobutylicum
Treponema pallidum
Chlamydia trachomatis
Campylobacter jejuni
Escherichia coli
Rhodopseudomonas palustris
Synechocystis sp. PCC 6803
Rhodopirellula baltica
100
89
100
75
21
28
14
14
93
0.2

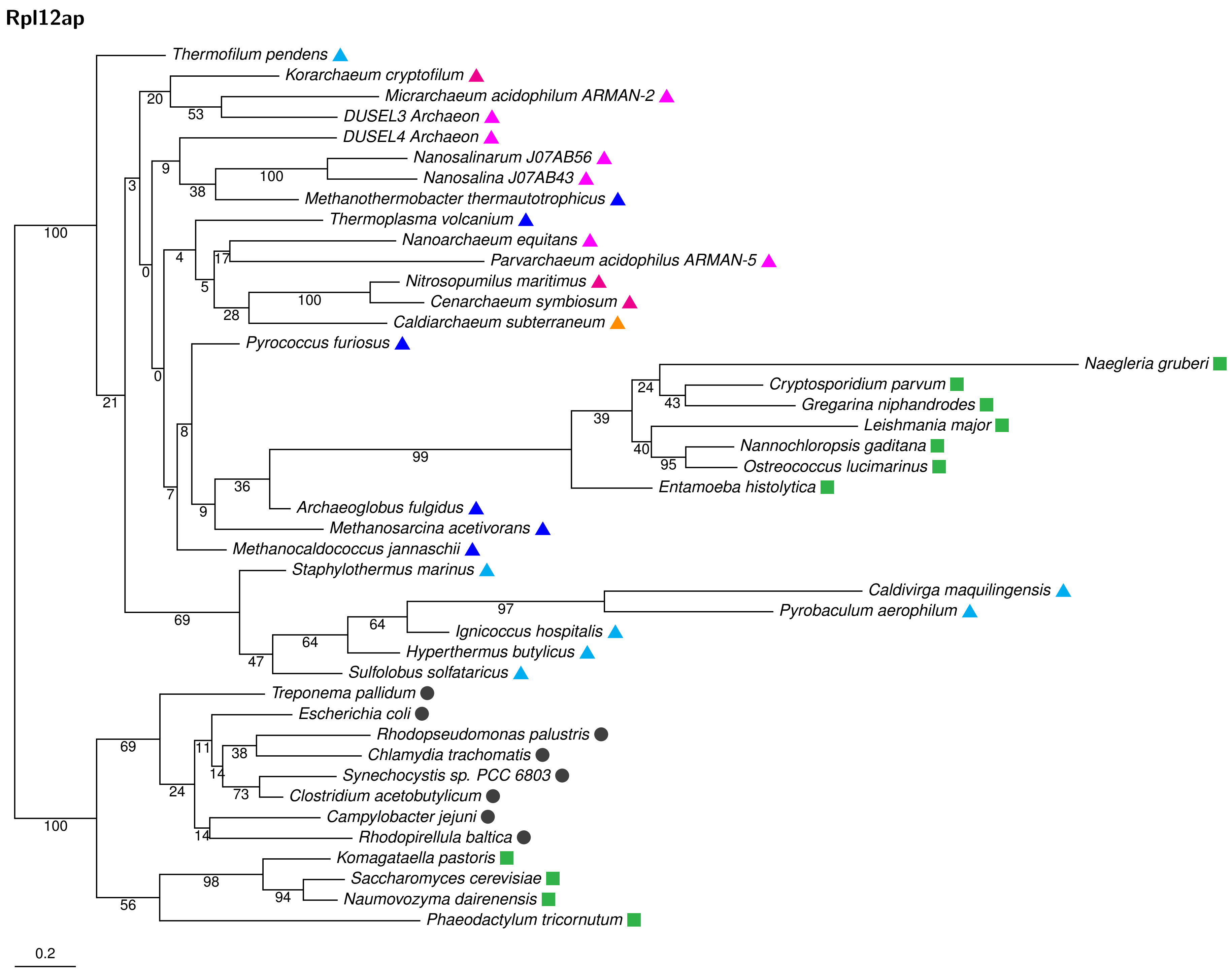
Rpl12ap
Thermofilum pendens
Korarchaeum cryptofilum
Micrarchaeum acidophilum ARMAN-2
DUSEL3 Archaeon
DUSEL4 Archaeon
Nanosalinarum J07AB56
Nanosalina J07AB43
Methanothermobacter thermautotrophicus
Thermoplasma volcanium
Nanoarchaeum equitans
Parvarchaeum acidophilus ARMAN-5
Nitrosopumilus maritimus
Cenarchaeum symbiosum
Caldiarchaeum subterraneum
Pyrococcus furiosus
Naegleria gruberi
Cryptosporidium parvum
Gregarina niphandrodes
Leishmania major
Nannochloropsis gaditana
Ostreococcus lucimarinus
Entamoeba histolytica
Archaeoglobus fulgidus
Methanosarcina acetivorans
Methanocaldococcus jannaschii
Staphylothermus marinus
Caldivirga maquilingensis
Pyrobaculum aerophilum
Ignicoccus hospitalis
Hyperthermus butylicus
Sulfolobus solfataricus
Treponema pallidum
Escherichia coli
Rhodopseudomonas palustris
Chlamydia trachomatis
Synechocystis sp. PCC 6803
Clostridium acetobutylicum
Campylobacter jejuni
Rhodopirellula baltica
Komagataella pastoris
Saccharomyces cerevisiae
Naumovozyma dairenensis
Phaeodactylum tricornutum
0.2

Rps11ap

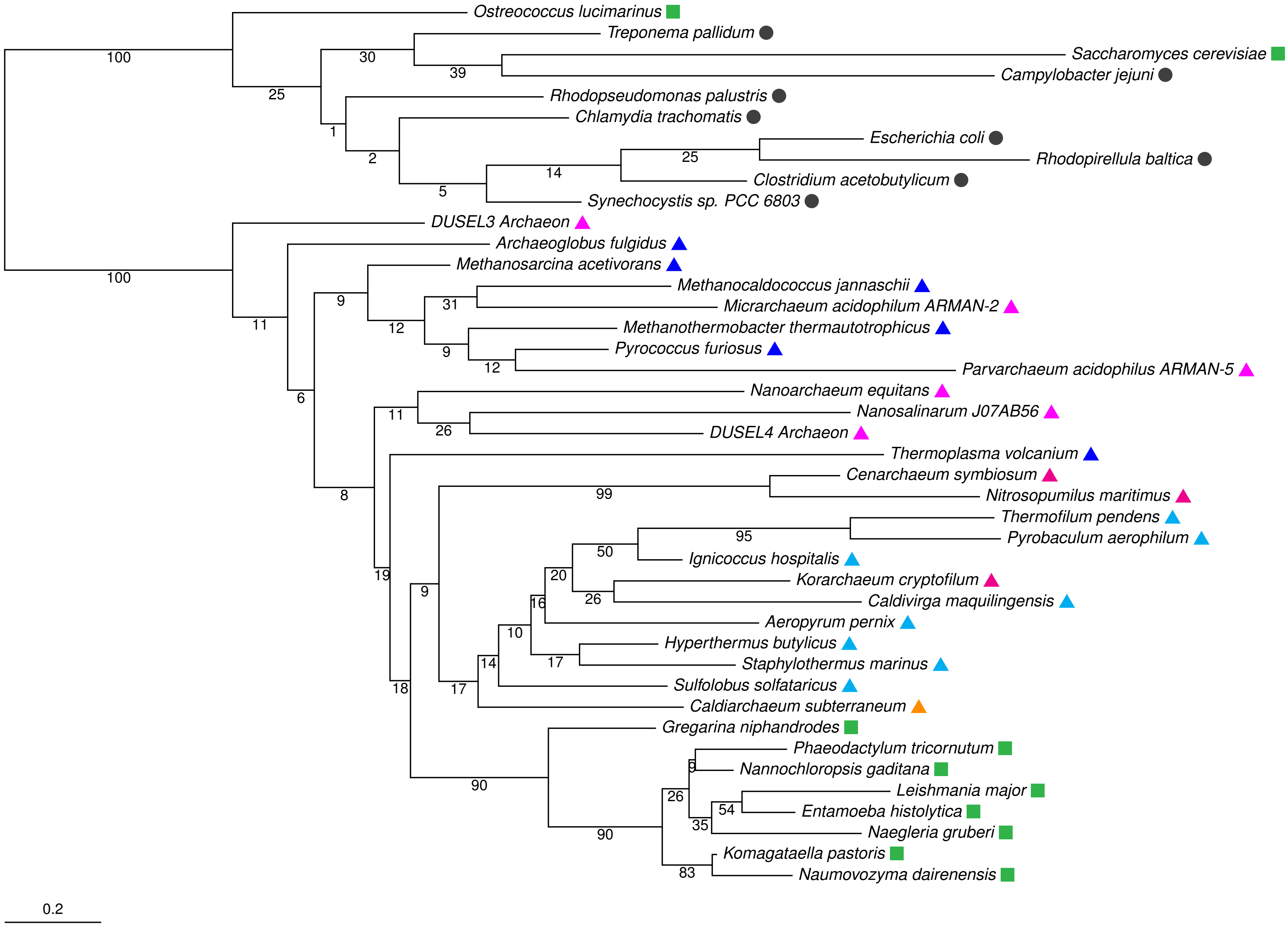

Ostreococcus lucimarinus
Treponema pallidum
Saccharomyces cerevisiae
Campylobacter jejuni
Rhodopseudomonas palustris
Chlamydia trachomatis
Escherichia coli
Rhodopirellula baltica
Clostridium acetobutylicum
Synechocystis sp. PCC 6803
DUSEL3 Archaeon
Archaeoglobus fulgidus
Methanosarcina acetivorans
Methanocaldococcus jannaschii
Micrarchaeum acidophilum ARMAN-2
Methanothermobacter thermautotrophicus
Pyrococcus furiosus
Parvarchaeum acidophilus ARMAN-5
Nanoarchaeum equitans
Nanosalinarum J07AB56
DUSEL4 Archaeon
Thermoplasma volcanium
Cenarchaeum symbiosum
Nitrosopumilus maritimus
Thermofilum pendens
Pyrobaculum aerophilum
Ignicoccus hospitalis
Korarchaeum cryptofilum
Caldivirga maquilingensis
Aeropyrum pernix
Hyperthermus butylicus
Staphylothermus marinus
Sulfolobus solfataricus
Caldiarchaeum subterraneum
Gregarina niphandrodes
Phaeodactylum tricornutum
Nannochloropsis gaditana
Leishmania major
Entamoeba histolytica
Naegleria gruberi
Komagataella pastoris
Naumovozyma dairenensis
100
100
25
30
39
1
2
5
14
25
11
6
9
12
31
9
12
8
11
26
19
18
9
99
95
50
20
26
16
10
17
14
17
90
90
26
54
35
83
0.2

Rpl2bp

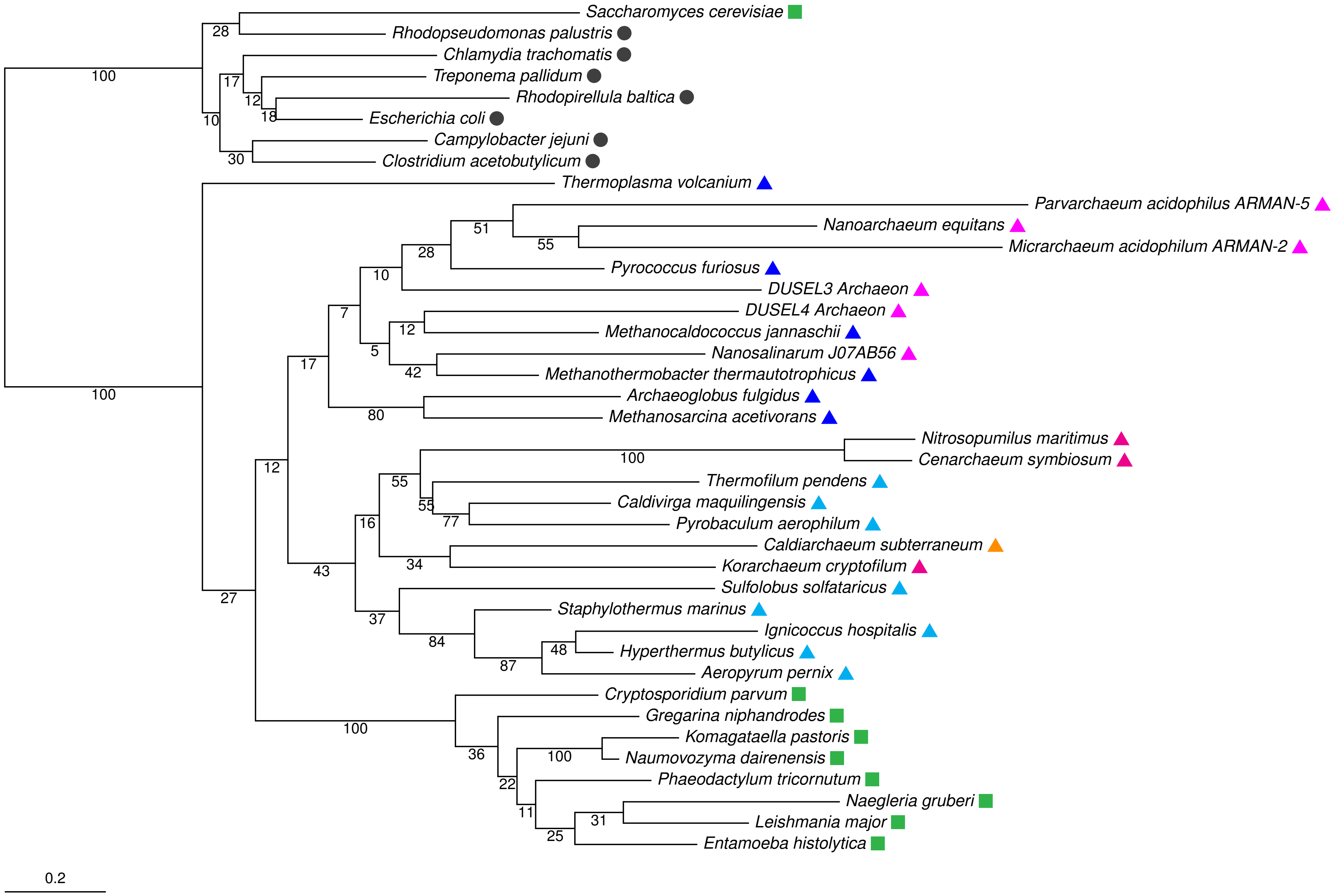
Saccharomyces cerevisiae
Rhodopseudomonas palustris
Chlamydia trachomatis
Treponema pallidum
Rhodopirellula baltica
Escherichia coli
Campylobacter jejuni
Clostridium acetobutylicum
Thermoplasma volcanium
Parvarchaeum acidophilus ARMAN-5
Nanoarchaeum equitans
Micrarchaeum acidophilum ARMAN-2
Pyrococcus furiosus
DUSEL3 Archaeon
DUSEL4 Archaeon
Methanocaldococcus jannaschii
Nanosalinarum J07AB56
Methanothermobacter thermautotrophicus
Archaeoglobus fulgidus
Methanosarcina acetivorans
Nitrosopumilus maritimus
Cenarchaeum symbiosum
Thermofilum pendens
Caldivirga maquilingensis
Pyrobaculum aerophilum
Caldiarchaeum subterraneum
Korarchaeum cryptofilum
Sulfolobus solfataricus
Staphylothermus marinus
Ignicoccus hospitalis
Hyperthermus butylicus
Aeropyrum pernix
Cryptosporidium parvum
Gregarina niphandrodes
Komagataella pastoris
Naumovozyma dairenensis
Phaeodactylum tricornutum
Naegleria gruberi
Leishmania major
Entamoeba histolytica
0.2

Mrps28p

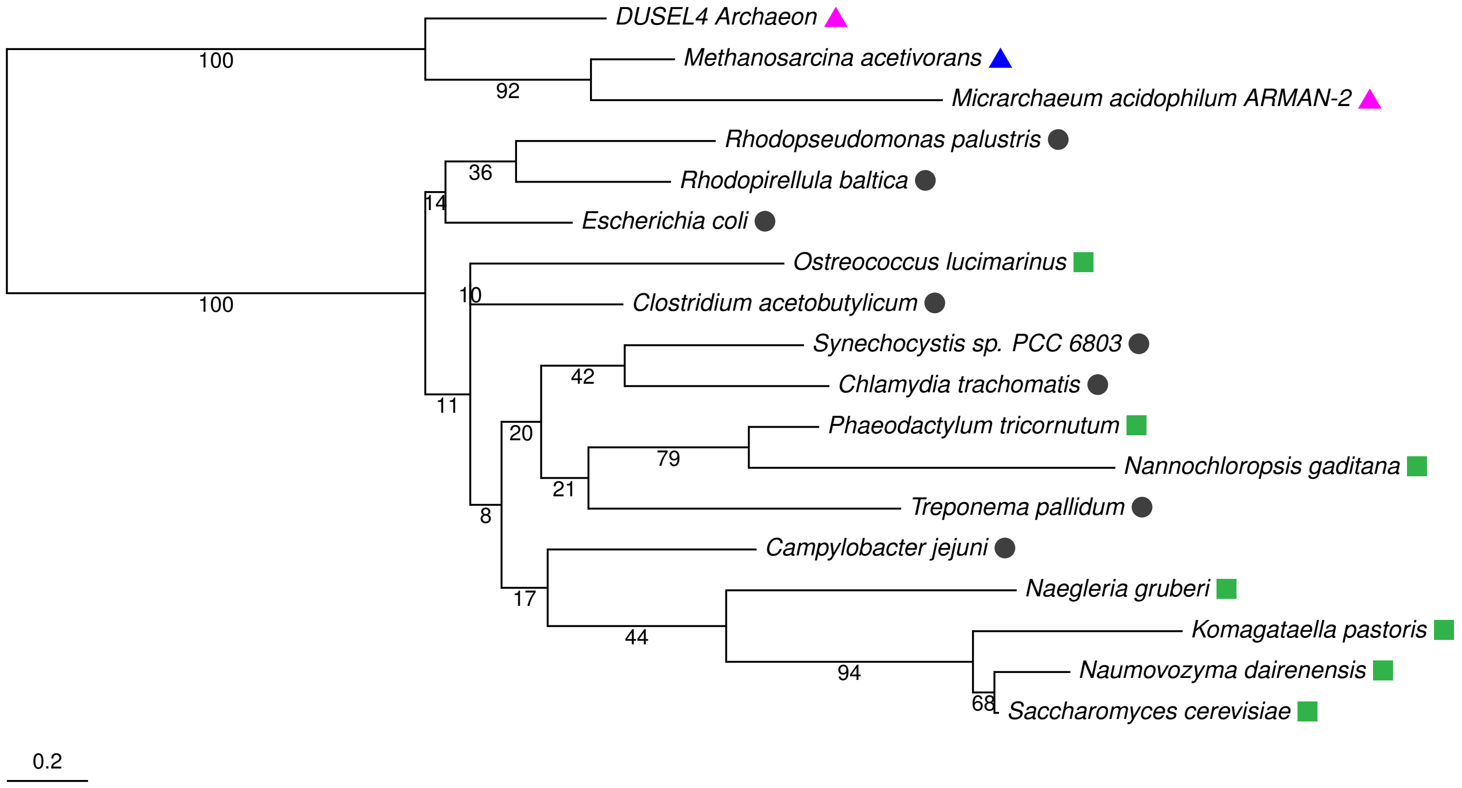
DUSEL4 Archaeon
Methanosarcina acetivorans
Micrarchaeum acidophilum ARMAN-2
Rhodopseudomonas palustris
Rhodopirellula baltica
Escherichia coli
Ostreococcus lucimarinus
Clostridium acetobutylicum
Synechocystis sp. PCC 6803
Chlamydia trachomatis
Phaeodactylum tricornutum
Nannochloropsis gaditana
Treponema pallidum
Campylobacter jejuni
Naegleria gruberi
Komagataella pastoris
Naumovozyma dairenensis
Saccharomyces cerevisiae
100
100
92
36
14
10
11
42
20
79
21
8
17
44
94
68
0.2

Rps16ap

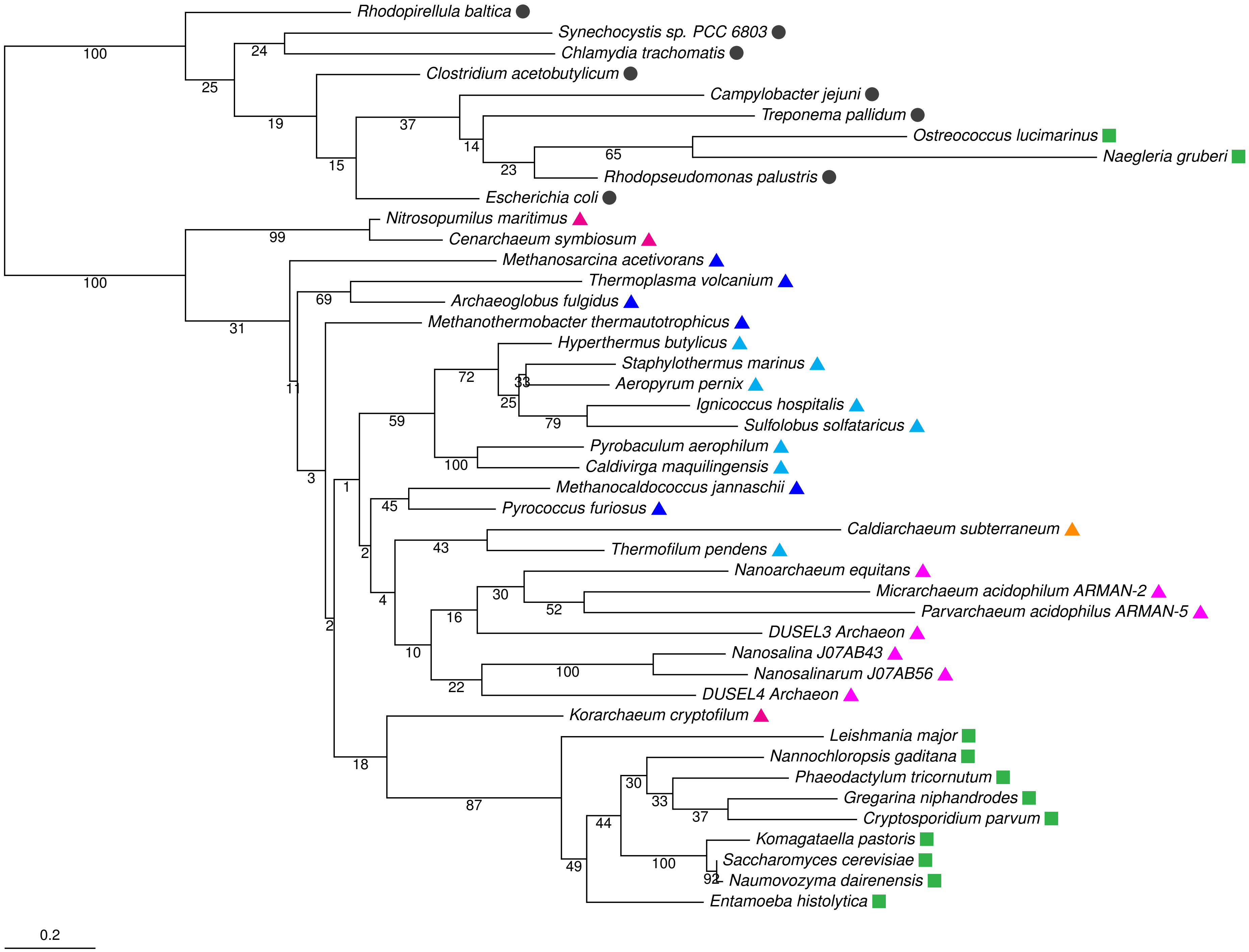

Rhodopirellula baltica
Synechocystis sp. PCC 6803
Chlamydia trachomatis
Clostridium acetobutylicum
Campylobacter jejuni
Treponema pallidum
Ostreococcus lucimarinus
Naegleria gruberi
Rhodopseudomonas palustris
Escherichia coli
Nitrosopumilus maritimus
Cenarchaeum symbiosum
Methanosarcina acetivorans
Thermoplasma volcanium
Archaeoglobus fulgidus
Methanothermobacter thermautotrophicus
Hyperthermus butylicus
Staphylothermus marinus
Aeropyrum pernix
Ignicoccus hospitalis
Sulfolobus solfataricus
Pyrobaculum aerophilum
Caldivirga maquilingensis
Methanocaldococcus jannaschii
Pyrococcus furiosus
Caldiarchaeum subterraneum
Thermofilum pendens
Nanoarchaeum equitans
Micrarchaeum acidophilum ARMAN-2
Parvarchaeum acidophilus ARMAN-5
DUSEL3 Archaeon
Nanosalina J07AB43
Nanosalinarum J07AB56
DUSEL4 Archaeon
Korarchaeum cryptofilum
Leishmania major
Nannochloropsis gaditana
Phaeodactylum tricornutum
Gregarina niphandrodes
Cryptosporidium parvum
Komagataella pastoris
Saccharomyces cerevisiae
Naumovozyma dairenensis
Entamoeba histolytica
100
25
24
19
37
15
14
23
65
100
99
31
69
11
3
59
72
33
25
79
100
1
45
2
43
4
30
52
16
10
100
22
2
18
87
49
44
30
33
37
100
92
0.2

Rpl11ap

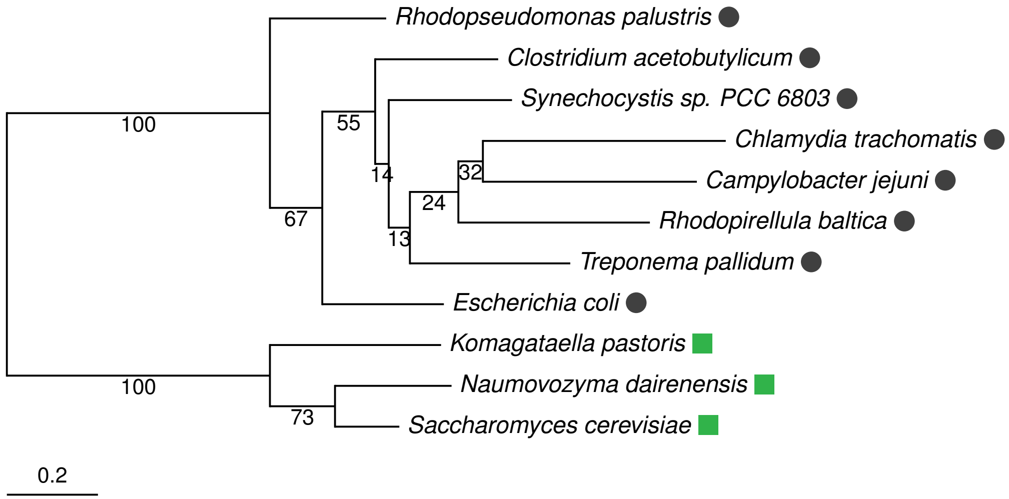

Rhodopseudomonas palustris
Clostridium acetobutylicum
Synechocystis sp. PCC 6803
Chlamydia trachomatis
Campylobacter jejuni
Rhodopirellula baltica
Treponema pallidum
Escherichia coli
Komagataella pastoris
Naumovozyma dairenensis
Saccharomyces cerevisiae
100
55
14
32
24
67
13
100
73
0.2

Rps5p

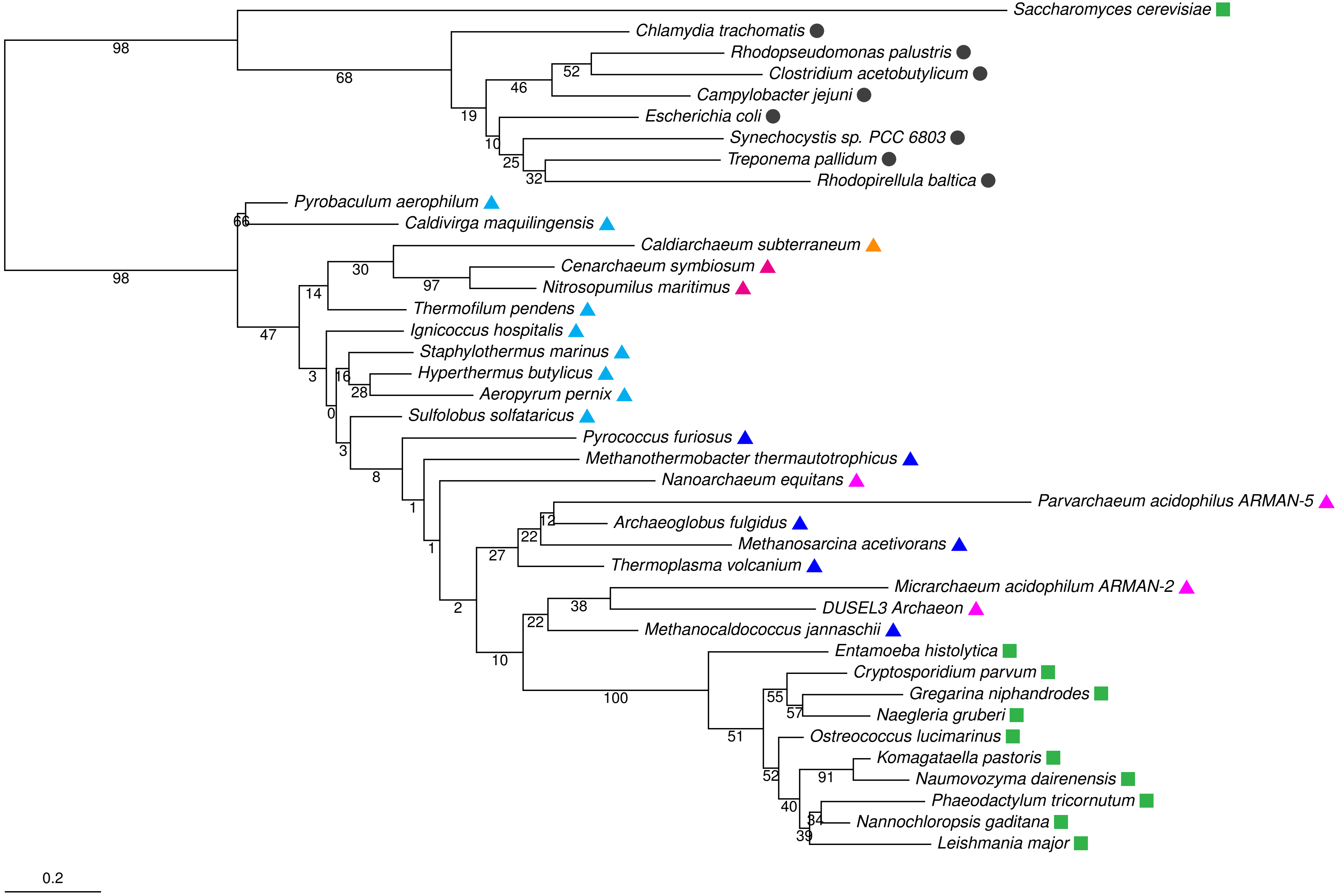
Saccharomyces cerevisiae
Chlamydia trachomatis
Rhodopseudomonas palustris
Clostridium acetobutylicum
Campylobacter jejuni
Escherichia coli
Synechocystis sp. PCC 6803
Treponema pallidum
Rhodopirellula baltica
Pyrobaculum aerophilum
Caldivirga maquilingensis
Caldiarchaeum subterraneum
Cenarchaeum symbiosum
Nitrosopumilus maritimus
Thermofilum pendens
Ignicoccus hospitalis
Staphylothermus marinus
Hyperthermus butylicus
Aeropyrum pernix
Sulfolobus solfataricus
Pyrococcus furiosus
Methanothermobacter thermautotrophicus
Nanoarchaeum equitans
Parvarchaeum acidophilus ARMAN-5
Archaeoglobus fulgidus
Methanosarcina acetivorans
Thermoplasma volcanium
Micrarchaeum acidophilum ARMAN-2
DUSEL3 Archaeon
Methanocaldococcus jannaschii
Entamoeba histolytica
Cryptosporidium parvum
Gregarina niphandrodes
Naegleria gruberi
Ostreococcus lucimarinus
Komagataella pastoris
Naumovozyma dairenensis
Phaeodactylum tricornutum
Nannochloropsis gaditana
Leishmania major
0.2

Fun12p

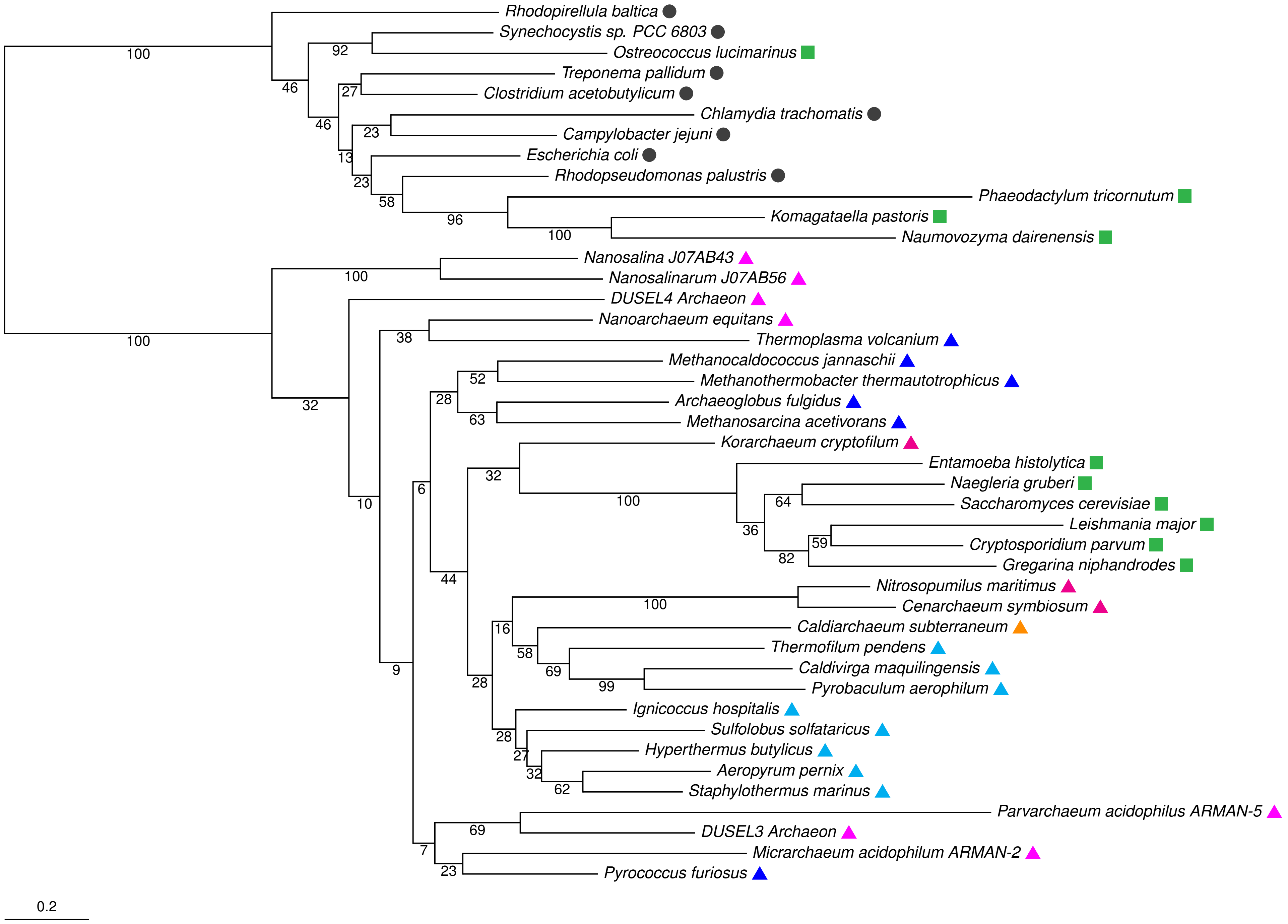

Rhodopirellula baltica
Synechocystis sp. PCC 6803
Ostreococcus lucimarinus
Treponema pallidum
Clostridium acetobutylicum
Chlamydia trachomatis
Campylobacter jejuni
Escherichia coli
Rhodopseudomonas palustris
Phaeodactylum tricornutum
Komagataella pastoris
Naumovozyma dairenensis
Nanosalina J07AB43
Nanosalinarum J07AB56
DUSEL4 Archaeon
Nanoarchaeum equitans
Thermoplasma volcanium
Methanocaldococcus jannaschii
Methanothermobacter thermautotrophicus
Archaeoglobus fulgidus
Methanosarcina acetivorans
Korarchaeum cryptofilum
Entamoeba histolytica
Naegleria gruberi
Saccharomyces cerevisiae
Leishmania major
Cryptosporidium parvum
Gregarina niphandrodes
Nitrosopumilus maritimus
Cenarchaeum symbiosum
Caldiarchaeum subterraneum
Thermofilum pendens
Caldivirga maquilingensis
Pyrobaculum aerophilum
Ignicoccus hospitalis
Sulfolobus solfataricus
Hyperthermus butylicus
Aeropyrum pernix
Staphylothermus marinus
Parvarchaeum acidophilus ARMAN-5
DUSEL3 Archaeon
Micrarchaeum acidophilum ARMAN-2
Pyrococcus furiosus
0.2

Rpl26bp

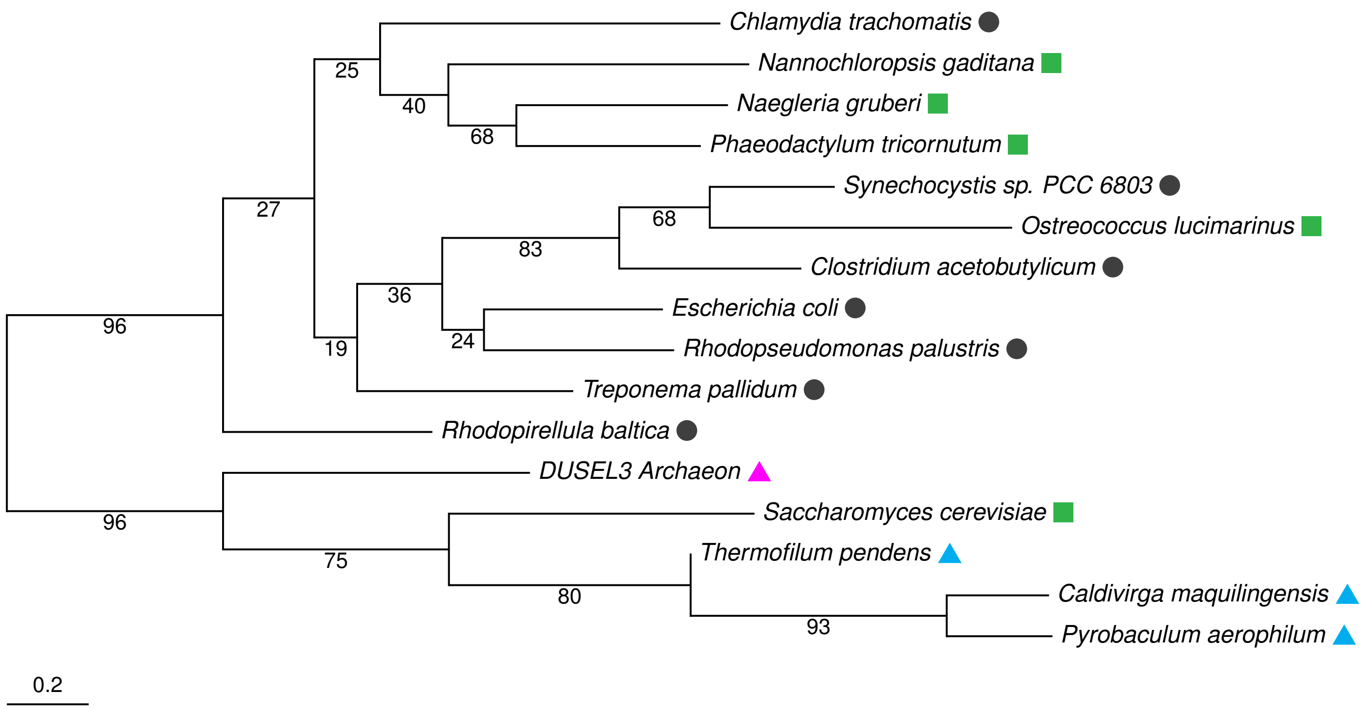

E. coli ribosomal gene L10

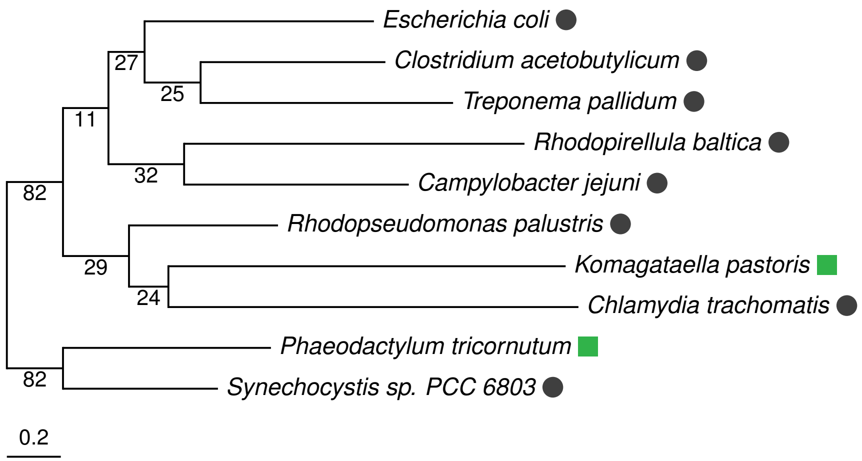

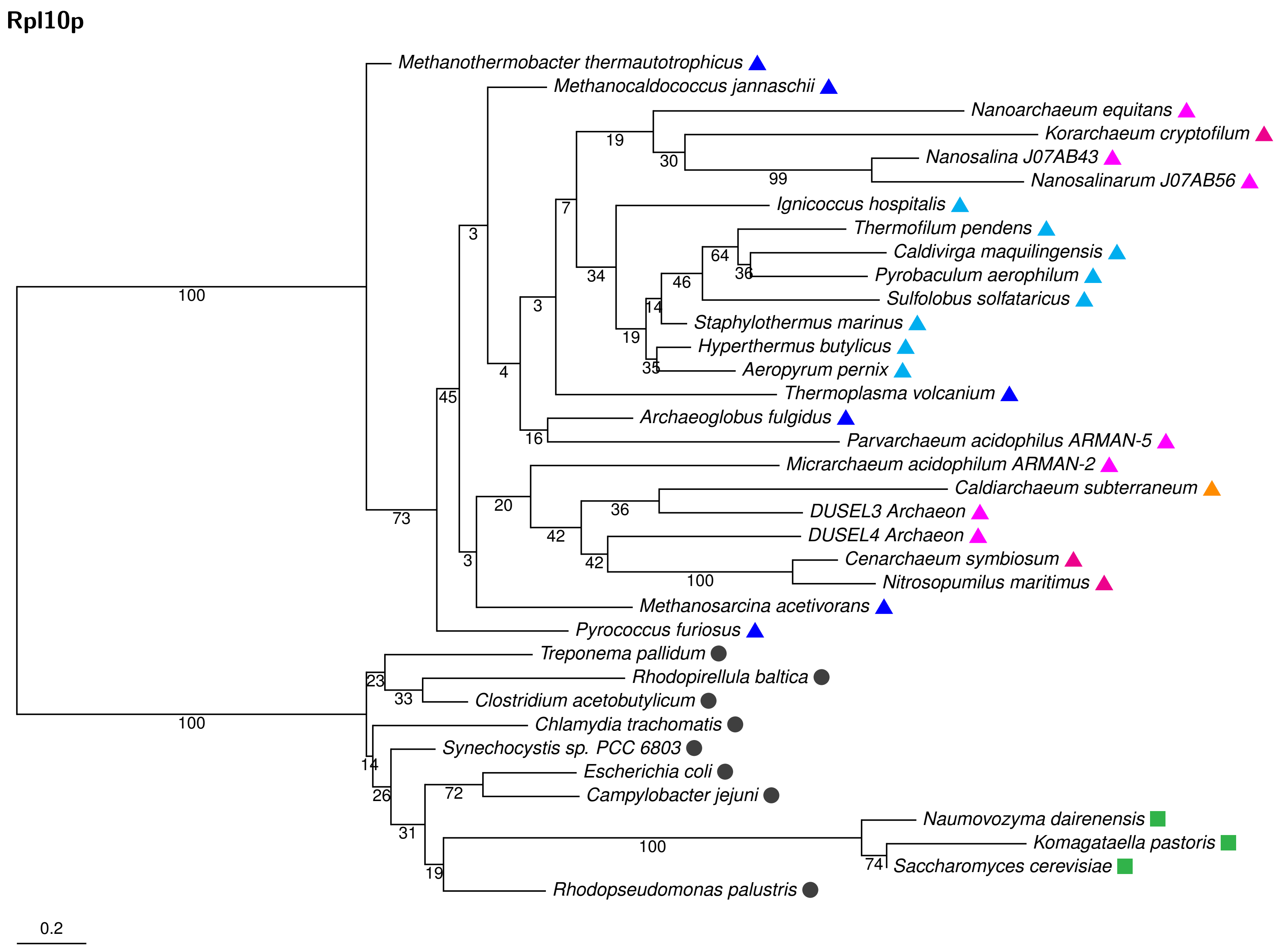
Rpl10p
Methanothermobacter thermautotrophicus
Methanocaldococcus jannaschii
Nanoarchaeum equitans
Korarchaeum cryptofilum
Nanosalina J07AB43
Nanosalinarum J07AB56
Ignicoccus hospitalis
Thermofilum pendens
Caldivirga maquilingensis
Pyrobaculum aerophilum
Sulfolobus solfataricus
Staphylothermus marinus
Hyperthermus butylicus
Aeropyrum pernix
Thermoplasma volcanium
Archaeoglobus fulgidus
Parvarchaeum acidophilus ARMAN-5
Micrarchaeum acidophilum ARMAN-2
Caldiarchaeum subterraneum
DUSEL3 Archaeon
DUSEL4 Archaeon
Cenarchaeum symbiosum
Nitrosopumilus maritimus
Methanosarcina acetivorans
Pyrococcus furiosus
Treponema pallidum
Rhodopirellula baltica
Clostridium acetobutylicum
Chlamydia trachomatis
Synechocystis sp. PCC 6803
Escherichia coli
Campylobacter jejuni
Naumovozyma dairenensis
Komagataella pastoris
Saccharomyces cerevisiae
Rhodopseudomonas palustris
100
100
73
45
3
3
4
3
7
19
30
99
34
64
36
46
14
19
35
16
20
36
42
42
100
3
23
33
14
26
31
72
19
100
74
0.2

Mrps8p

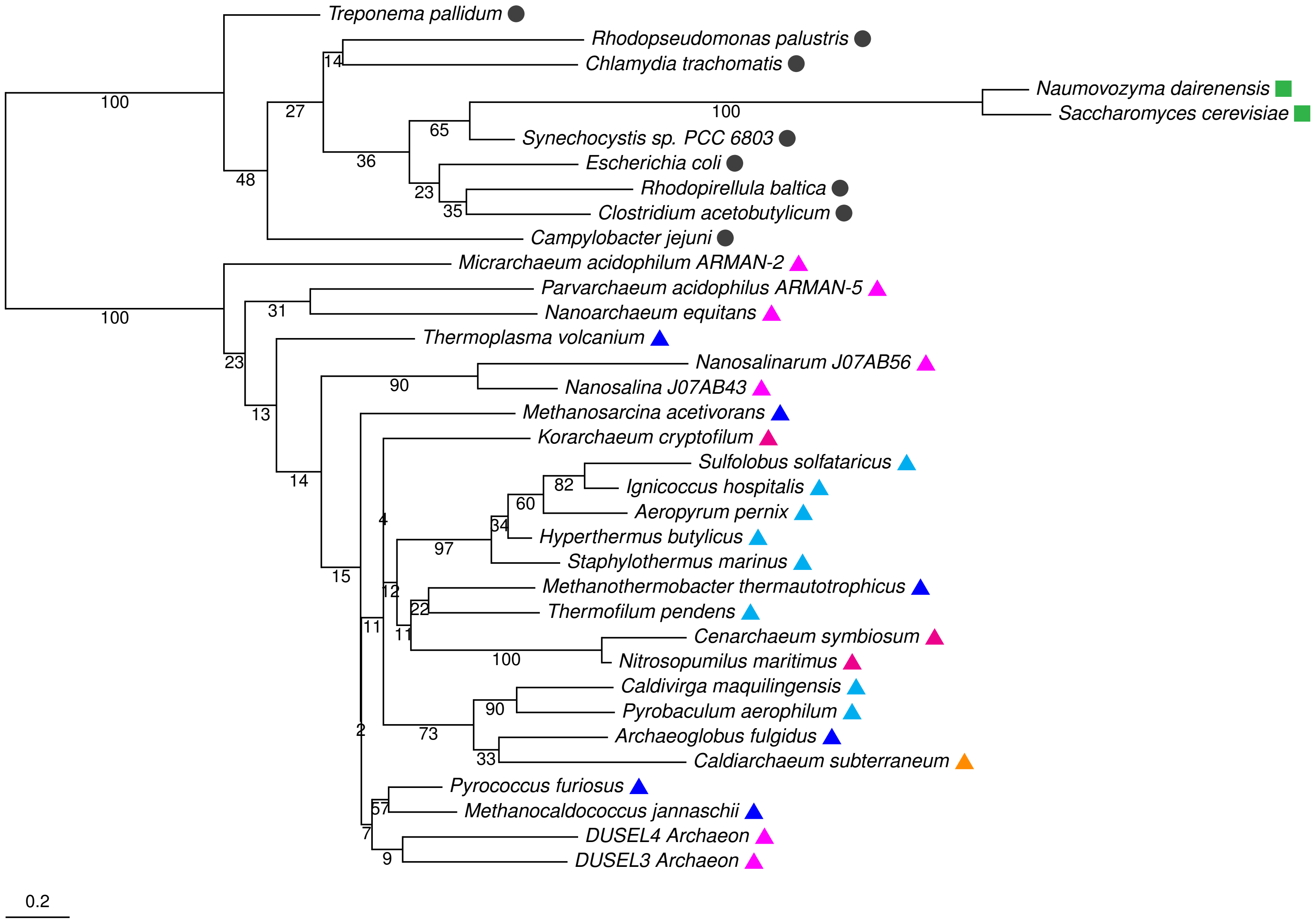
Treponema pallidum
Rhodopseudomonas palustris
Chlamydia trachomatis
Naumovozyma dairenensis
Saccharomyces cerevisiae
Synechocystis sp. PCC 6803
Escherichia coli
Rhodopirellula baltica
Clostridium acetobutylicum
Campylobacter jejuni
Micrarchaeum acidophilum ARMAN-2
Parvarchaeum acidophilus ARMAN-5
Nanoarchaeum equitans
Thermoplasma volcanium
Nanosalinarum J07AB56
Nanosalina J07AB43
Methanosarcina acetivorans
Korarchaeum cryptofilum
Sulfolobus solfataricus
Ignicoccus hospitalis
Aeropyrum pernix
Hyperthermus butylicus
Staphylothermus marinus
Methanothermobacter thermautotrophicus
Thermofilum pendens
Cenarchaeum symbiosum
Nitrosopumilus maritimus
Caldivirga maquilingensis
Pyrobaculum aerophilum
Archaeoglobus fulgidus
Caldiarchaeum subterraneum
Pyrococcus furiosus
Methanocaldococcus jannaschii
DUSEL4 Archaeon
DUSEL3 Archaeon
0.2

Rpl3p

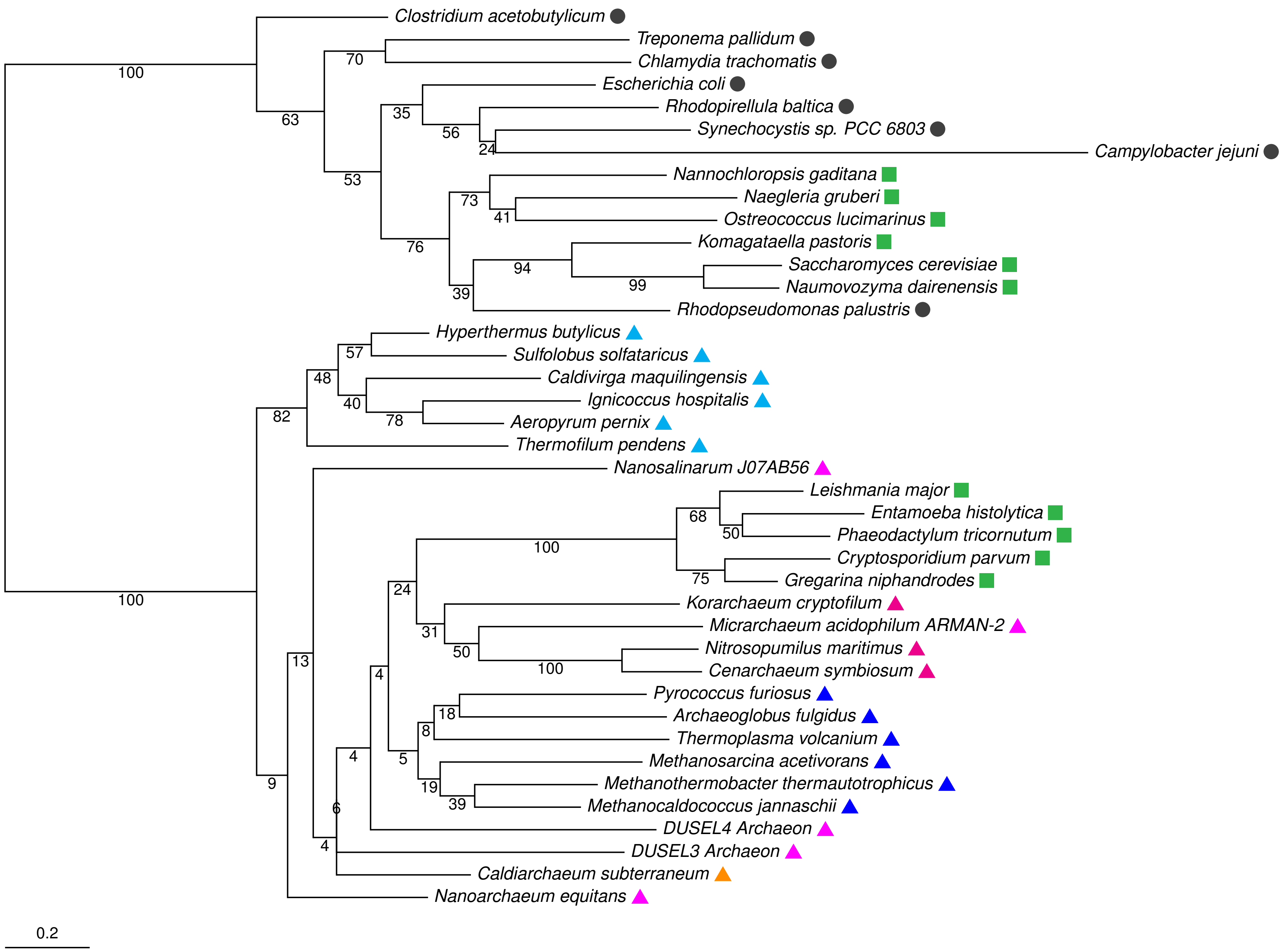
Clostridium acetobutylicum
Treponema pallidum
Chlamydia trachomatis
Escherichia coli
Rhodopirellula baltica
Synechocystis sp. PCC 6803
Campylobacter jejuni
Nannochloropsis gaditana
Naegleria gruberi
Ostreococcus lucimarinus
Komagataella pastoris
Saccharomyces cerevisiae
Naumovozyma dairenensis
Rhodopseudomonas palustris
Hyperthermus butylicus
Sulfolobus solfataricus
Caldivirga maquilingensis
Ignicoccus hospitalis
Aeropyrum pernix
Thermofilum pendens
Nanosalinarum J07AB56
Leishmania major
Entamoeba histolytica
Phaeodactylum tricornutum
Cryptosporidium parvum
Gregarina niphandrodes
Korarchaeum cryptofilum
Micrarchaeum acidophilum ARMAN-2
Nitrosopumilus maritimus
Cenarchaeum symbiosum
Pyrococcus furiosus
Archaeoglobus fulgidus
Thermoplasma volcanium
Methanosarcina acetivorans
Methanothermobacter thermautotrophicus
Methanocaldococcus jannaschii
DUSEL4 Archaeon
DUSEL3 Archaeon
Caldiarchaeum subterraneum
Nanoarchaeum equitans
0.2

Rps18bp

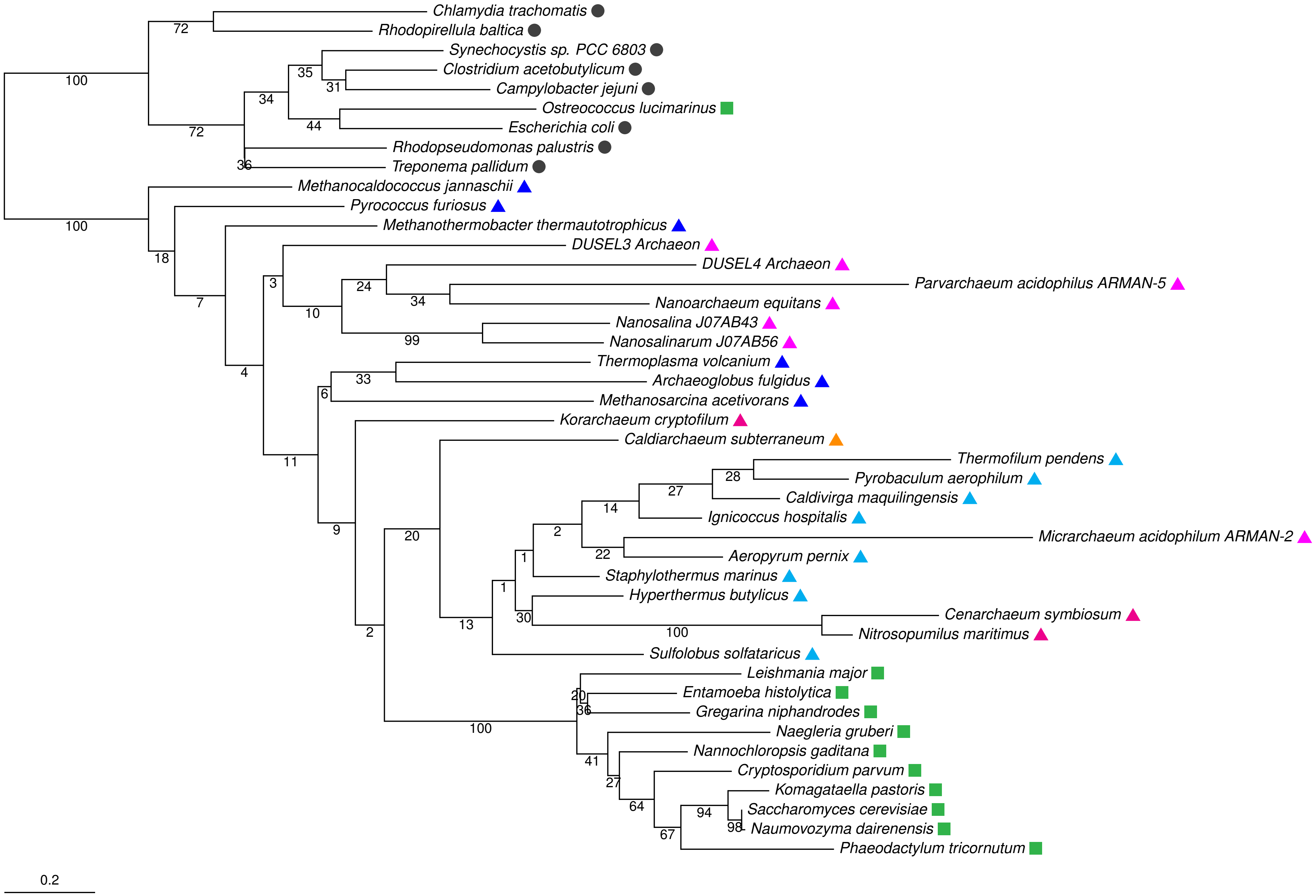

Chlamydia trachomatis
Rhodopirellula baltica
Synechocystis sp. PCC 6803
Clostridium acetobutylicum
Campylobacter jejuni
Ostreococcus lucimarinus
Escherichia coli
Rhodopseudomonas palustris
Treponema pallidum
Methanocaldococcus jannaschii
Pyrococcus furiosus
Methanothermobacter thermautotrophicus
DUSEL3 Archaeon
DUSEL4 Archaeon
Parvarchaeum acidophilus ARMAN-5
Nanoarchaeum equitans
Nanosalina J07AB43
Nanosalinarum J07AB56
Thermoplasma volcanium
Archaeoglobus fulgidus
Methanosarcina acetivorans
Korarchaeum cryptofilum
Caldiarchaeum subterraneum
Thermofilum pendens
Pyrobaculum aerophilum
Caldivirga maquilingensis
Ignicoccus hospitalis
Micrarchaeum acidophilum ARMAN-2
Aeropyrum pernix
Staphylothermus marinus
Hyperthermus butylicus
Cenarchaeum symbiosum
Nitrosopumilus maritimus
Sulfolobus solfataricus
Leishmania major
Entamoeba histolytica
Gregarina niphandrodes
Naegleria gruberi
Nannochloropsis gaditana
Cryptosporidium parvum
Komagataella pastoris
Saccharomyces cerevisiae
Naumovozyma dairenensis
Phaeodactylum tricornutum
0.2

Frs2p

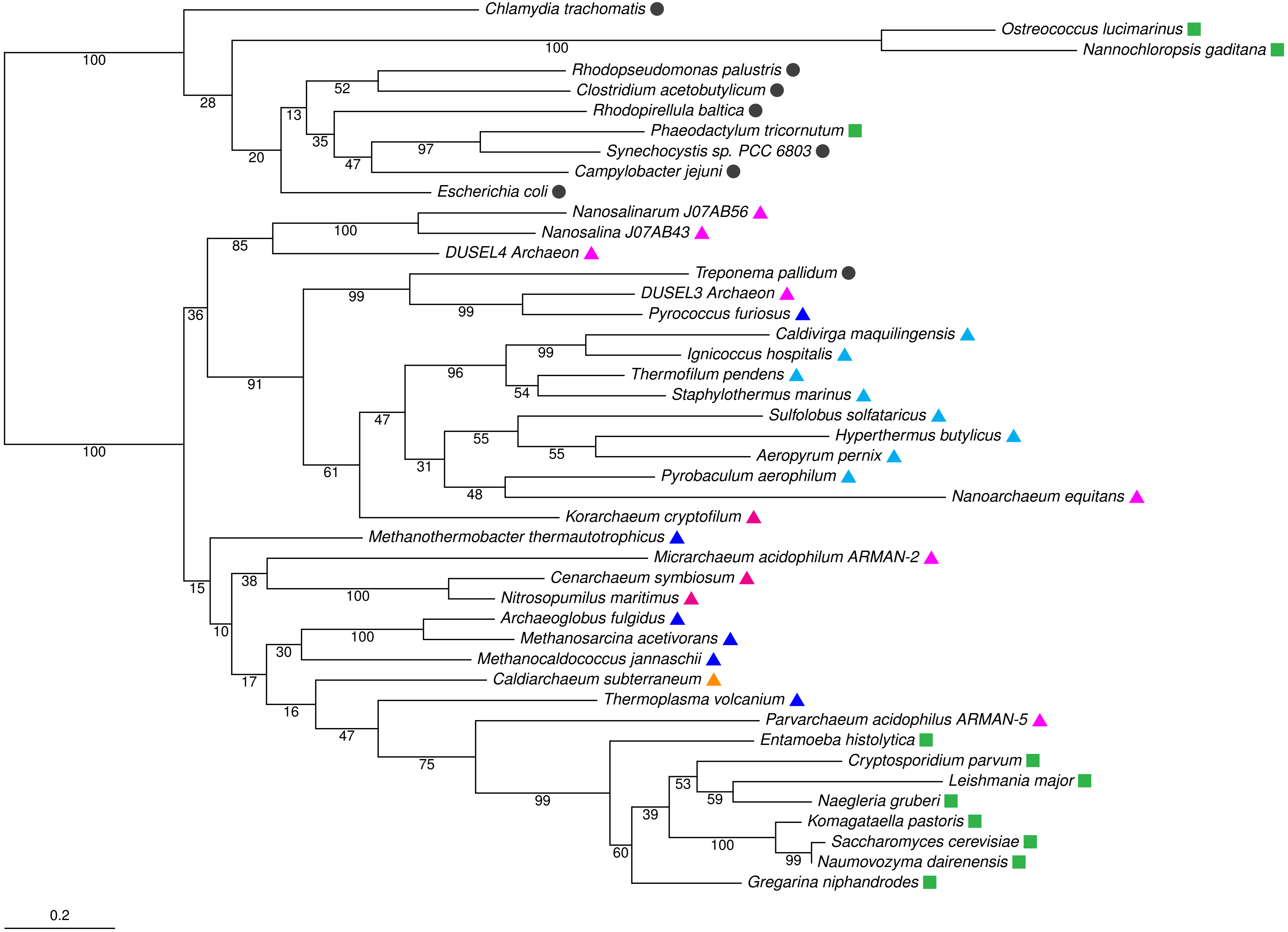
Chlamydia trachomatis
Ostreococcus lucimarinus
Nannochloropsis gaditana
Rhodopseudomonas palustris
Clostridium acetobutylicum
Rhodopirellula baltica
Phaeodactylum tricornutum
Synechocystis sp. PCC 6803
Campylobacter jejuni
Escherichia coli
Nanosalinarum J07AB56
Nanosalina J07AB43
DUSEL4 Archaeon
Treponema pallidum
DUSEL3 Archaeon
Pyrococcus furiosus
Caldivirga maquilingensis
Ignicoccus hospitalis
Thermofilum pendens
Staphylothermus marinus
Sulfolobus solfataricus
Hyperthermus butylicus
Aeropyrum pernix
Pyrobaculum aerophilum
Nanoarchaeum equitans
Korarchaeum cryptofilum
Methanothermobacter thermautotrophicus
Micrarchaeum acidophilum ARMAN-2
Cenarchaeum symbiosum
Nitrosopumilus maritimus
Archaeoglobus fulgidus
Methanosarcina acetivorans
Methanocaldococcus jannaschii
Caldiarchaeum subterraneum
Thermoplasma volcanium
Parvarchaeum acidophilus ARMAN-5
Entamoeba histolytica
Cryptosporidium parvum
Leishmania major
Naegleria gruberi
Komagataella pastoris
Saccharomyces cerevisiae
Naumovozyma dairenensis
Gregarina niphandrodes
0.2

Mrpl10p

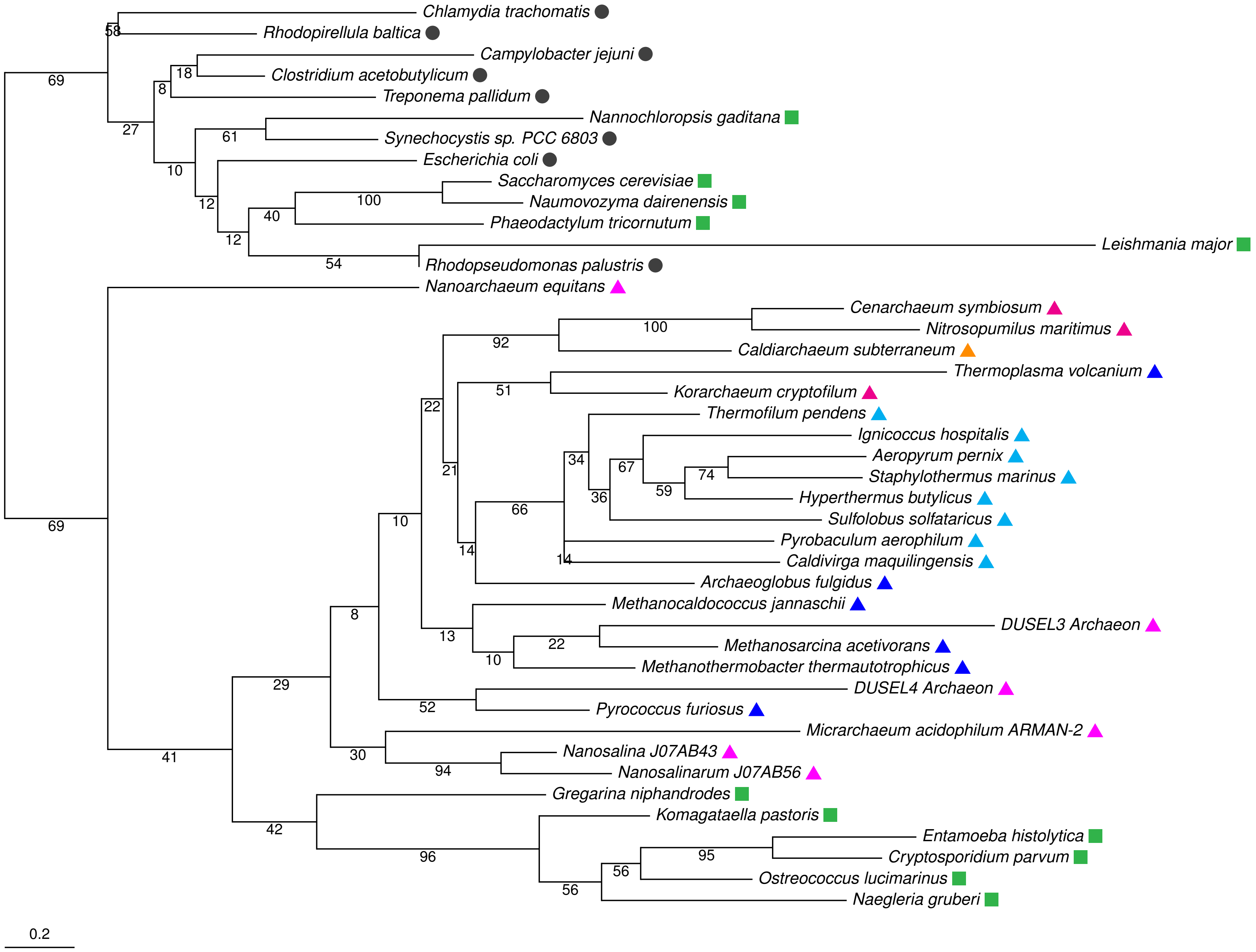

Chlamydia trachomatis
Rhodopirellula baltica
Campylobacter jejuni
Clostridium acetobutylicum
Treponema pallidum
Nannochloropsis gaditana
Synechocystis sp. PCC 6803
Escherichia coli
Saccharomyces cerevisiae
Naumovozyma dairenensis
Phaeodactylum tricornutum
Leishmania major
Rhodopseudomonas palustris
Nanoarchaeum equitans
Cenarchaeum symbiosum
Nitrosopumilus maritimus
Caldiarchaeum subterraneum
Thermoplasma volcanium
Korarchaeum cryptofilum
Thermofilum pendens
Ignicoccus hospitalis
Aeropyrum pernix
Staphylothermus marinus
Hyperthermus butylicus
Sulfolobus solfataricus
Pyrobaculum aerophilum
Caldivirga maquilingensis
Archaeoglobus fulgidus
Methanocaldococcus jannaschii
DUSEL3 Archaeon
Methanosarcina acetivorans
Methanothermobacter thermautotrophicus
DUSEL4 Archaeon
Pyrococcus furiosus
Micrarchaeum acidophilum ARMAN-2
Nanosalina J07AB43
Nanosalinarum J07AB56
Gregarina niphandrodes
Komagataella pastoris
Entamoeba histolytica
Cryptosporidium parvum
Ostreococcus lucimarinus
Naegleria gruberi
0.2

E. coli queuine tRNA-ribosyltransferase

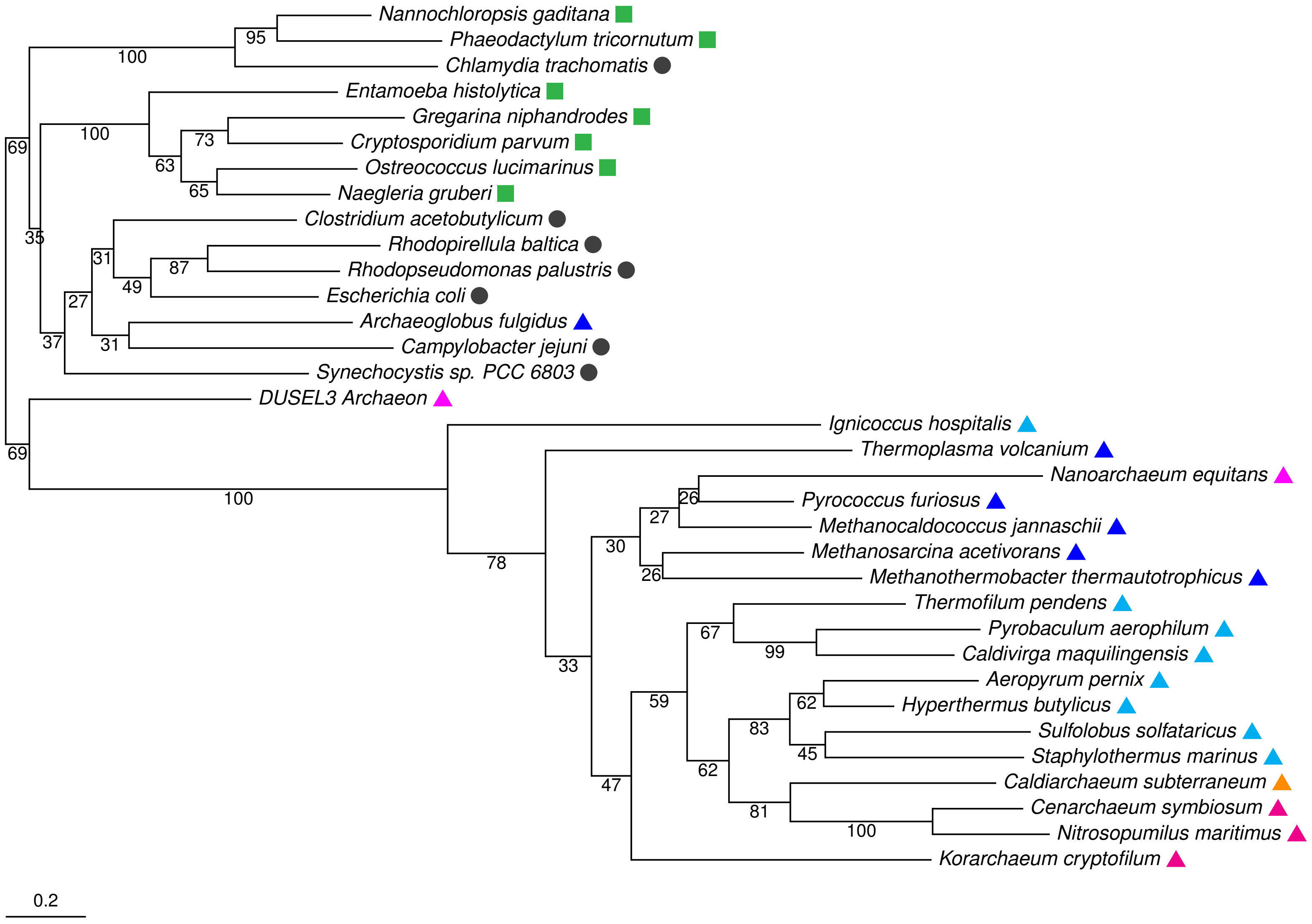

Rnh201p

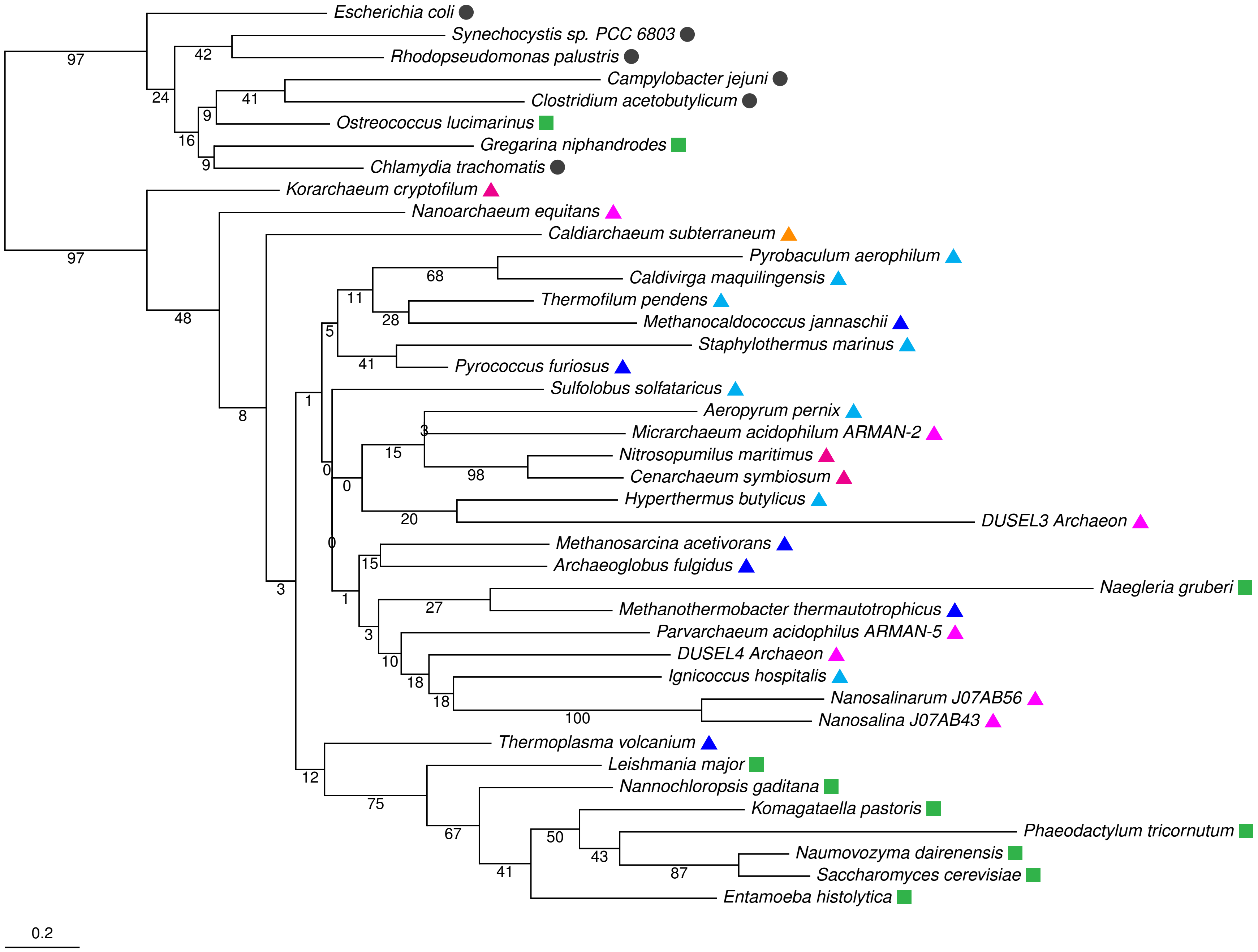
Escherichia coli
Synechocystis sp. PCC 6803
Rhodopseudomonas palustris
Campylobacter jejuni
Clostridium acetobutylicum
Ostreococcus lucimarinus
Gregarina niphandrodes
Chlamydia trachomatis
Korarchaeum cryptofilum
Nanoarchaeum equitans
Caldiarchaeum subterraneum
Pyrobaculum aerophilum
Caldivirga maquilingensis
Thermofilum pendens
Methanocaldococcus jannaschii
Staphylothermus marinus
Pyrococcus furiosus
Sulfolobus solfataricus
Aeropyrum pernix
Micrarchaeum acidophilum ARMAN-2
Nitrosopumilus maritimus
Cenarchaeum symbiosum
Hyperthermus butylicus
DUSEL3 Archaeon
Methanosarcina acetivorans
Archaeoglobus fulgidus
Naegleria gruberi
Methanothermobacter thermautotrophicus
Parvarchaeum acidophilus ARMAN-5
DUSEL4 Archaeon
Ignicoccus hospitalis
Nanosalinarum J07AB56
Nanosalina J07AB43
Thermoplasma volcanium
Leishmania major
Nannochloropsis gaditana
Komagataella pastoris
Phaeodactylum tricornutum
Naumovozyma dairenensis
Saccharomyces cerevisiae
Entamoeba histolytica
0.2

Genes without contamination

Rps3p

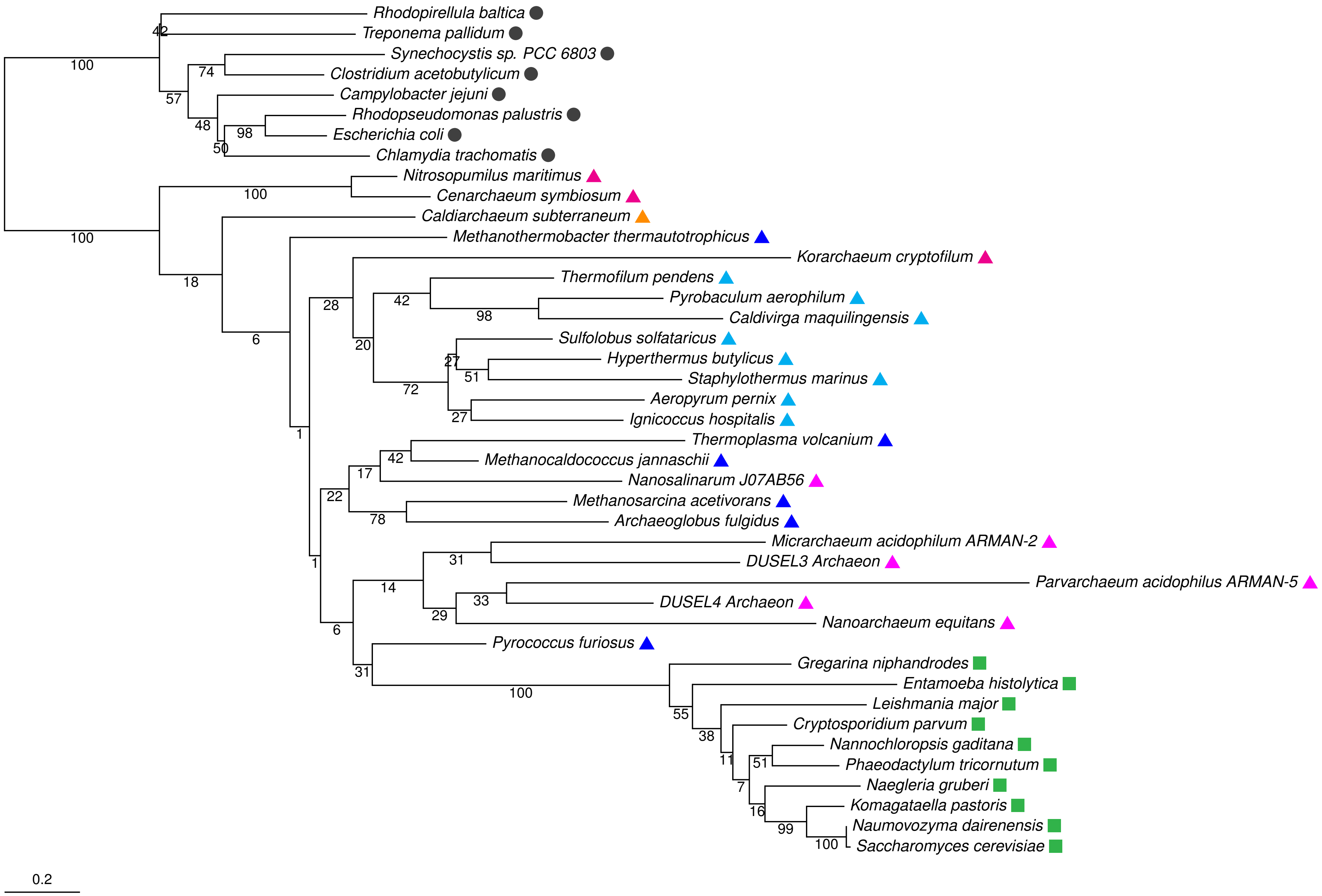

Rpl1ap

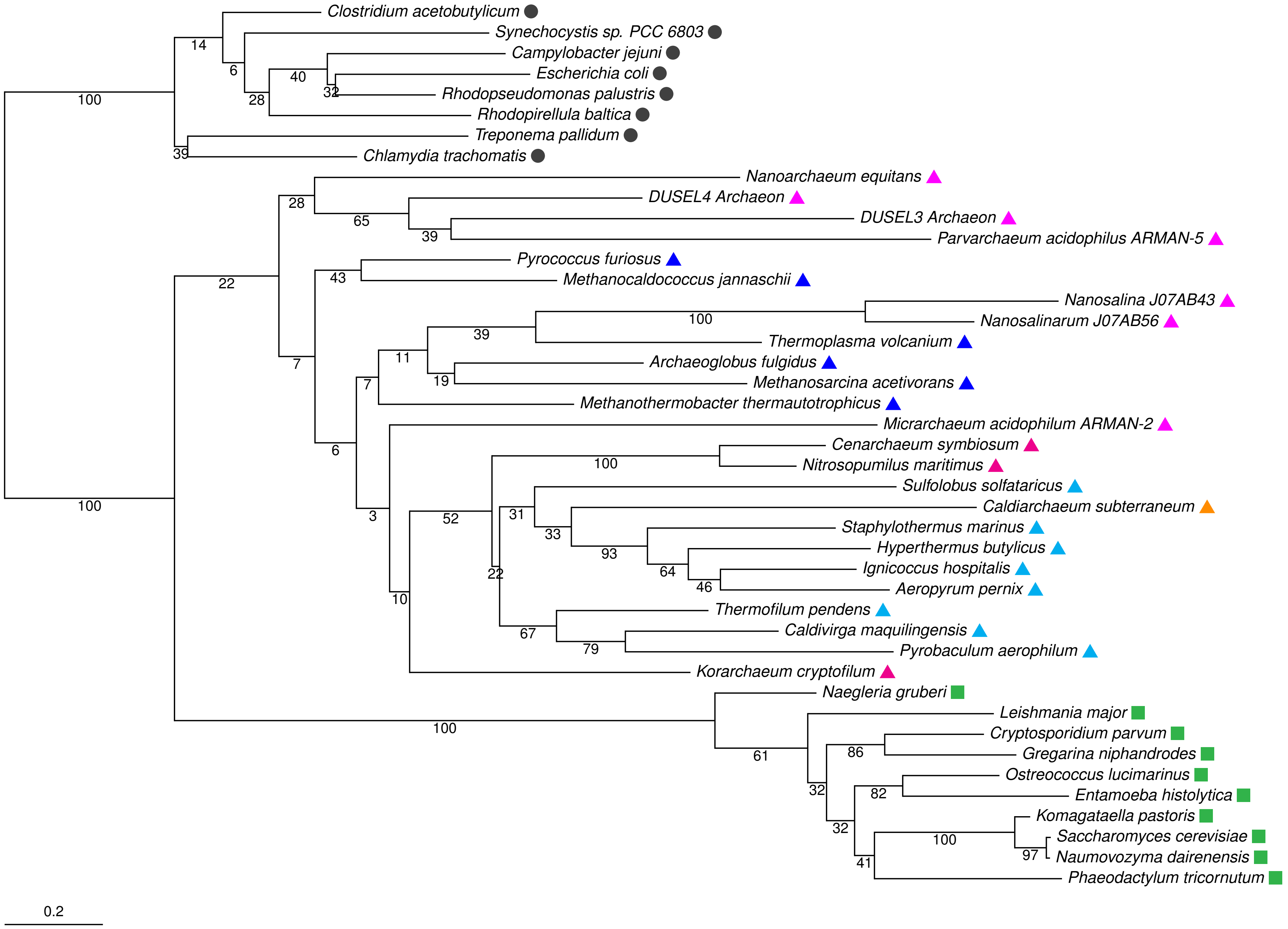

Clostridium acetobutylicum
Synechocystis sp. PCC 6803
Campylobacter jejuni
Escherichia coli
Rhodopseudomonas palustris
Rhodopirellula baltica
Treponema pallidum
Chlamydia trachomatis
Nanoarchaeum equitans
DUSEL4 Archaeon
DUSEL3 Archaeon
Parvarchaeum acidophilus ARMAN-5
Pyrococcus furiosus
Methanocaldococcus jannaschii
Nanosalina J07AB43
Nanosalinarum J07AB56
Thermoplasma volcanium
Archaeoglobus fulgidus
Methanosarcina acetivorans
Methanothermobacter thermautotrophicus
Micrarchaeum acidophilum ARMAN-2
Cenarchaeum symbiosum
Nitrosopumilus maritimus
Sulfolobus solfataricus
Caldiarchaeum subterraneum
Staphylothermus marinus
Hyperthermus butylicus
Ignicoccus hospitalis
Aeropyrum pernix
Thermofilum pendens
Caldivirga maquilingensis
Pyrobaculum aerophilum
Korarchaeum cryptofilum
Naegleria gruberi
Leishmania major
Cryptosporidium parvum
Gregarina niphandrodes
Ostreococcus lucimarinus
Entamoeba histolytica
Komagataella pastoris
Saccharomyces cerevisiae
Naumovozyma dairenensis
Phaeodactylum tricornutum
0.2

Rps15p

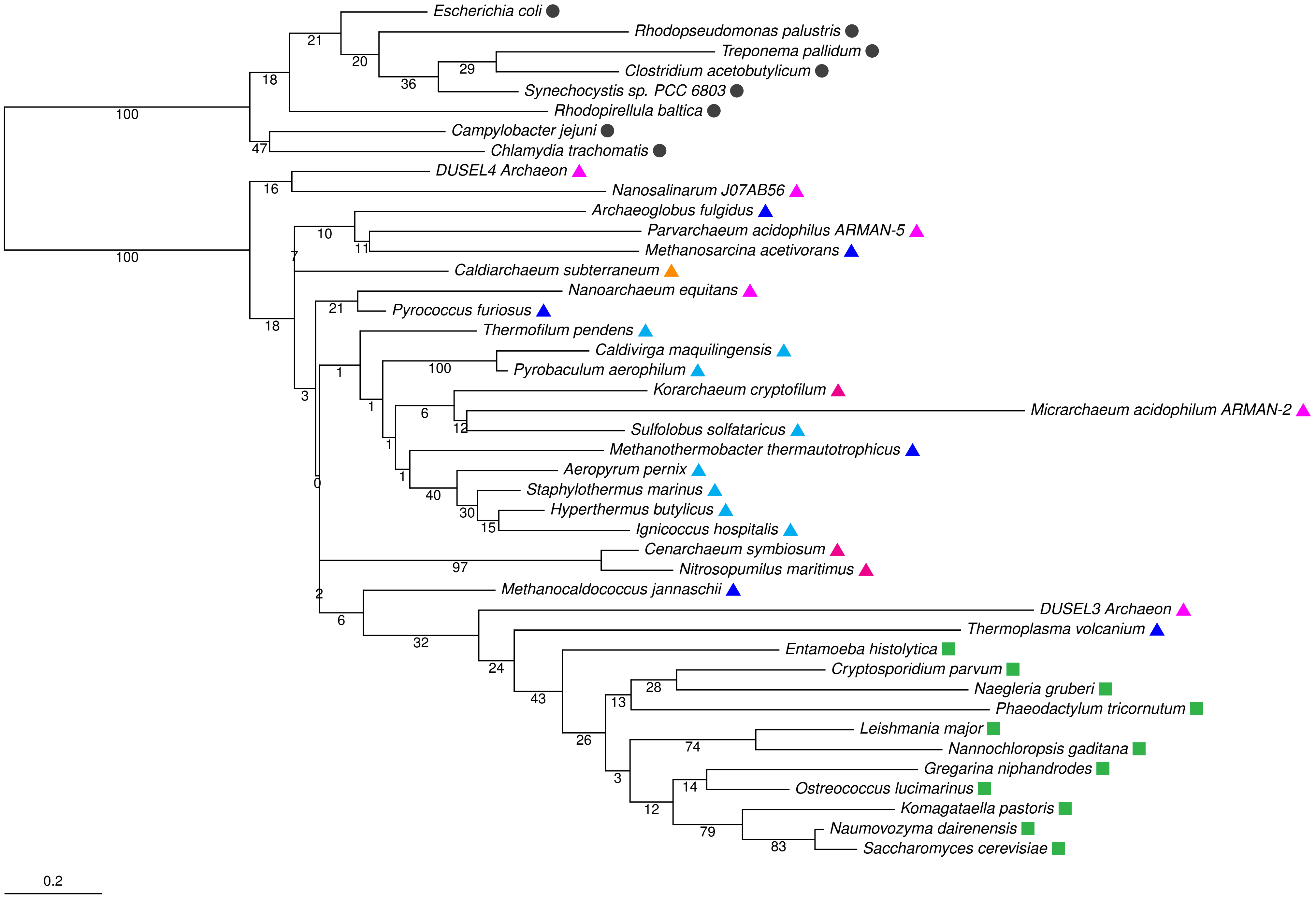

Escherichia coli
Rhodopseudomonas palustris
Treponema pallidum
Clostridium acetobutylicum
Synechocystis sp. PCC 6803
Rhodopirellula baltica
Campylobacter jejuni
Chlamydia trachomatis
DUSEL4 Archaeon
Nanosalinarum J07AB56
Archaeoglobus fulgidus
Parvarchaeum acidophilus ARMAN-5
Methanosarcina acetivorans
Caldiarchaeum subterraneum
Nanoarchaeum equitans
Pyrococcus furiosus
Thermofilum pendens
Caldivirga maquilingensis
Pyrobaculum aerophilum
Korarchaeum cryptofilum
Micrarchaeum acidophilum ARMAN-2
Sulfolobus solfataricus
Methanothermobacter thermautotrophicus
Aeropyrum pernix
Staphylothermus marinus
Hyperthermus butylicus
Ignicoccus hospitalis
Cenarchaeum symbiosum
Nitrosopumilus maritimus
Methanocaldococcus jannaschii
DUSEL3 Archaeon
Thermoplasma volcanium
Entamoeba histolytica
Cryptosporidium parvum
Naegleria gruberi
Phaeodactylum tricornutum
Leishmania major
Nannochloropsis gaditana
Gregarina niphandrodes
Ostreococcus lucimarinus
Komagataella pastoris
Naumovozyma dairenensis
Saccharomyces cerevisiae
0.2

Srp54p

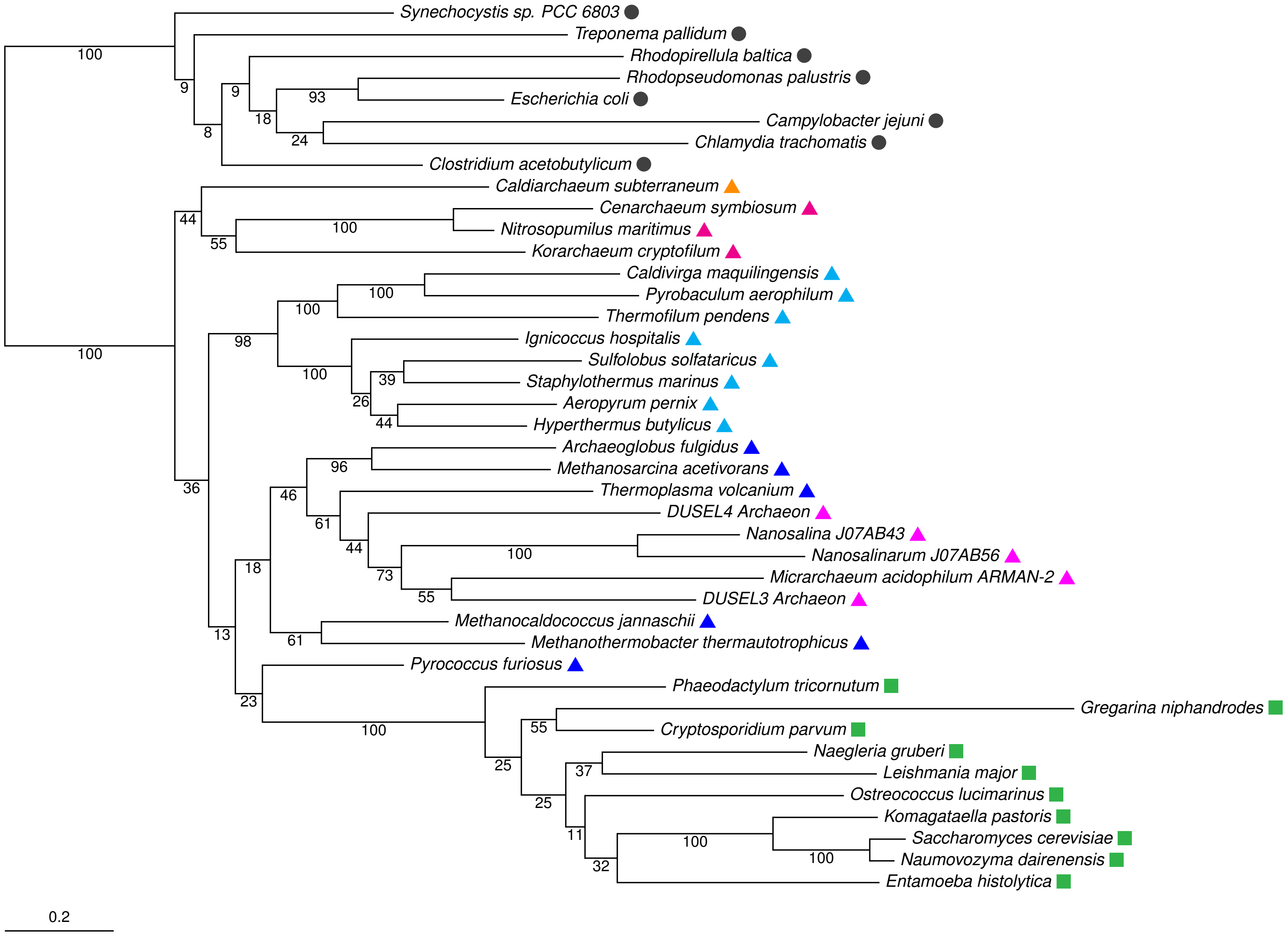
Synechocystis sp. PCC 6803
Treponema pallidum
Rhodopirellula baltica
Rhodopseudomonas palustris
Escherichia coli
Campylobacter jejuni
Chlamydia trachomatis
Clostridium acetobutylicum
Caldiarchaeum subterraneum
Cenarchaeum symbiosum
Nitrosopumilus maritimus
Korarchaeum cryptofilum
Caldivirga maquilingensis
Pyrobaculum aerophilum
Thermofilum pendens
Ignicoccus hospitalis
Sulfolobus solfataricus
Staphylothermus marinus
Aeropyrum pernix
Hyperthermus butylicus
Archaeoglobus fulgidus
Methanosarcina acetivorans
Thermoplasma volcanium
DUSEL4 Archaeon
Nanosalina J07AB43
Nanosalinarum J07AB56
Micrarchaeum acidophilum ARMAN-2
DUSEL3 Archaeon
Methanocaldococcus jannaschii
Methanothermobacter thermautotrophicus
Pyrococcus furiosus
Phaeodactylum tricornutum
Gregarina niphandrodes
Cryptosporidium parvum
Naegleria gruberi
Leishmania major
Ostreococcus lucimarinus
Komagataella pastoris
Saccharomyces cerevisiae
Naumovozyma dairenensis
Entamoeba histolytica
0.2

Rpl4bp

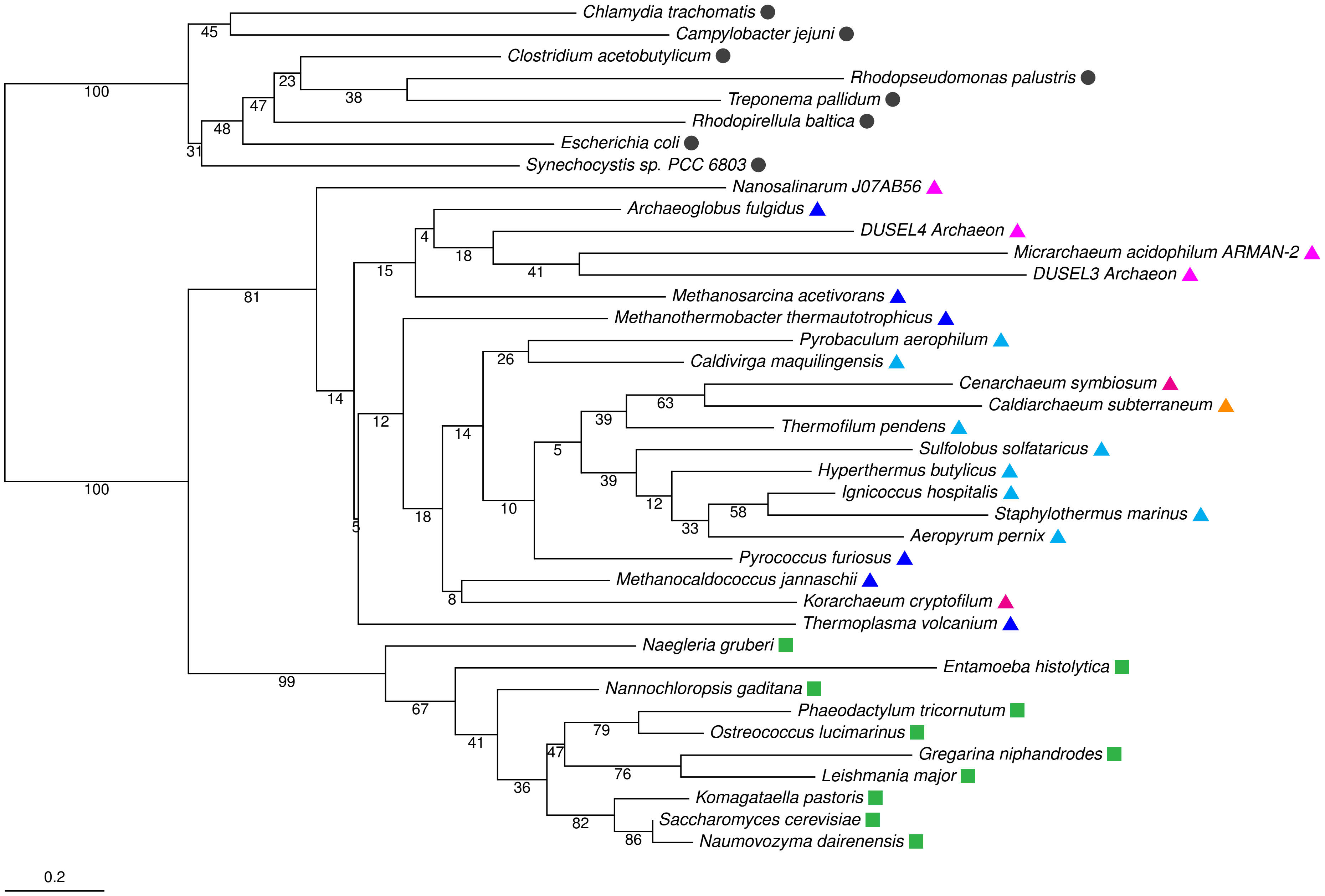
Chlamydia trachomatis
Campylobacter jejuni
Clostridium acetobutylicum
Rhodopseudomonas palustris
Treponema pallidum
Rhodopirellula baltica
Escherichia coli
Synechocystis sp. PCC 6803
Nanosalinarum J07AB56
Archaeoglobus fulgidus
DUSEL4 Archaeon
Micrarchaeum acidophilum ARMAN-2
DUSEL3 Archaeon
Methanosarcina acetivorans
Methanothermobacter thermautotrophicus
Pyrobaculum aerophilum
Caldivirga maquilingensis
Cenarchaeum symbiosum
Caldiarchaeum subterraneum
Thermofilum pendens
Sulfolobus solfataricus
Hyperthermus butylicus
Ignicoccus hospitalis
Staphylothermus marinus
Aeropyrum pernix
Pyrococcus furiosus
Methanocaldococcus jannaschii
Korarchaeum cryptofilum
Thermoplasma volcanium
Naegleria gruberi
Entamoeba histolytica
Nannochloropsis gaditana
Phaeodactylum tricornutum
Ostreococcus lucimarinus
Gregarina niphandrodes
Leishmania major
Komagataella pastoris
Saccharomyces cerevisiae
Naumovozyma dairenensis
0.2

Ura8p

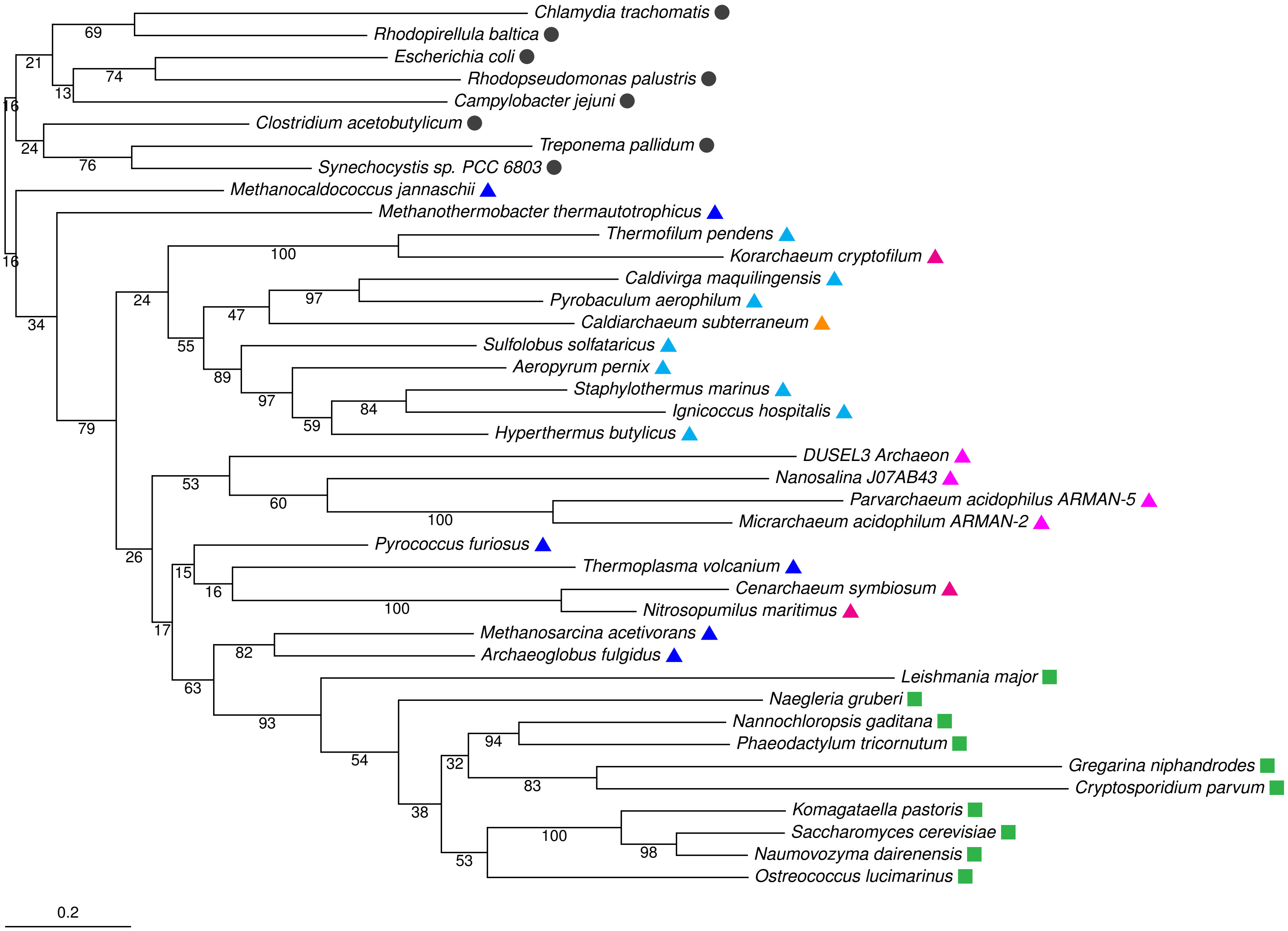

Chlamydia trachomatis
Rhodopirellula baltica
Escherichia coli
Rhodopseudomonas palustris
Campylobacter jejuni
Clostridium acetobutylicum
Treponema pallidum
Synechocystis sp. PCC 6803
Methanocaldococcus jannaschii
Methanothermobacter thermautotrophicus
Thermofilum pendens
Korarchaeum cryptofilum
Caldivirga maquilingensis
Pyrobaculum aerophilum
Caldiarchaeum subterraneum
Sulfolobus solfataricus
Aeropyrum pernix
Staphylothermus marinus
Ignicoccus hospitalis
Hyperthermus butylicus
DUSEL3 Archaeon
Nanosalina J07AB43
Parvarchaeum acidophilus ARMAN-5
Micrarchaeum acidophilum ARMAN-2
Pyrococcus furiosus
Thermoplasma volcanium
Cenarchaeum symbiosum
Nitrosopumilus maritimus
Methanosarcina acetivorans
Archaeoglobus fulgidus
Leishmania major
Naegleria gruberi
Nannochloropsis gaditana
Phaeodactylum tricornutum
Gregarina niphandrodes
Cryptosporidium parvum
Komagataella pastoris
Saccharomyces cerevisiae
Naumovozyma dairenensis
Ostreococcus lucimarinus
0.2

Rps14bp

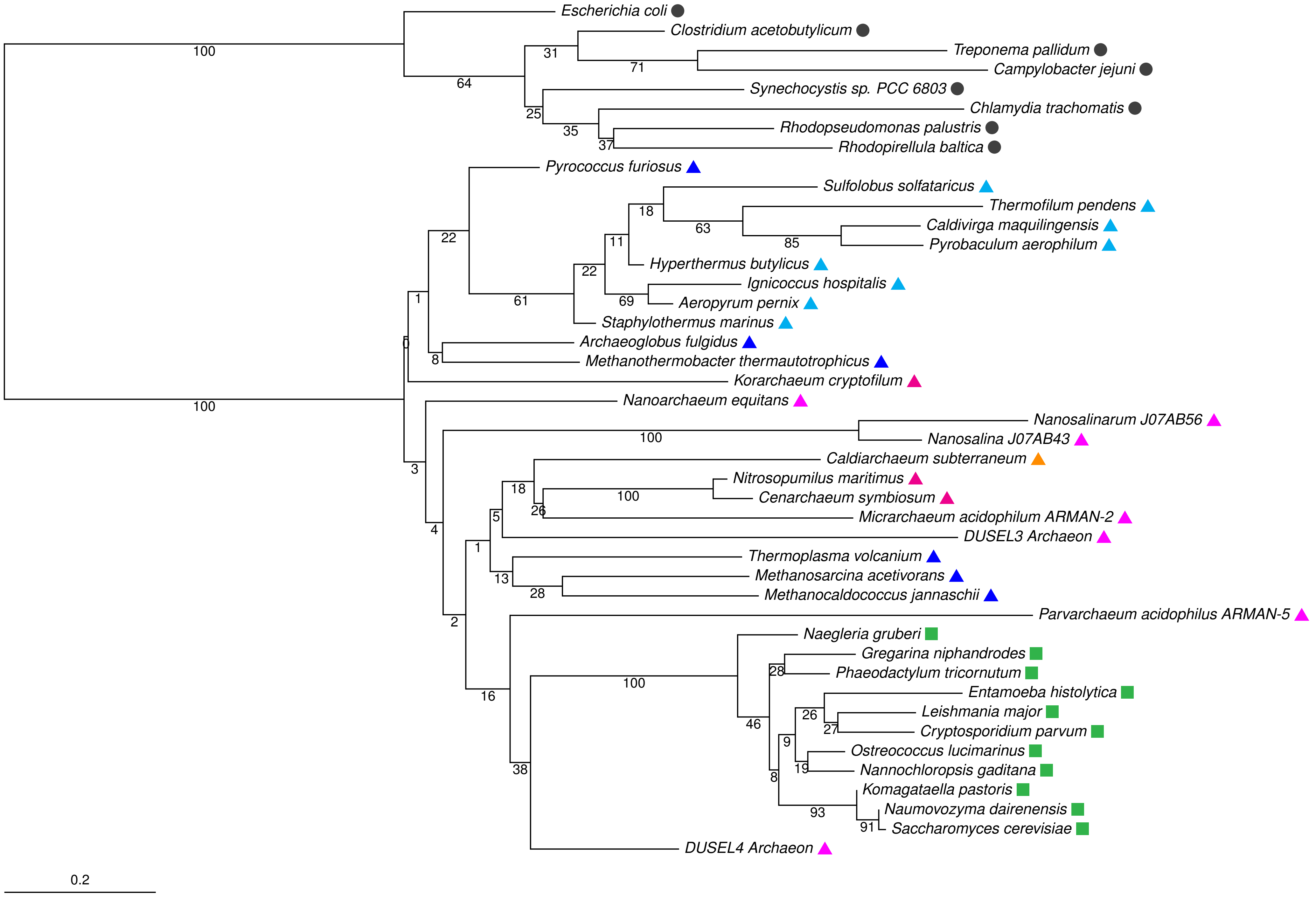

Escherichia coli
Clostridium acetobutylicum
Treponema pallidum
Campylobacter jejuni
Synechocystis sp. PCC 6803
Chlamydia trachomatis
Rhodopseudomonas palustris
Rhodopirellula baltica
Pyrococcus furiosus
Sulfolobus solfataricus
Thermofilum pendens
Caldivirga maquilingensis
Pyrobaculum aerophilum
Hyperthermus butylicus
Ignicoccus hospitalis
Aeropyrum pernix
Staphylothermus marinus
Archaeoglobus fulgidus
Methanothermobacter thermautotrophicus
Korarchaeum cryptofilum
Nanoarchaeum equitans
Nanosalinarum J07AB56
Nanosalina J07AB43
Caldiarchaeum subterraneum
Nitrosopumilus maritimus
Cenarchaeum symbiosum
Micrarchaeum acidophilum ARMAN-2
DUSEL3 Archaeon
Thermoplasma volcanium
Methanosarcina acetivorans
Methanocaldococcus jannaschii
Parvarchaeum acidophilus ARMAN-5
Naegleria gruberi
Gregarina niphandrodes
Phaeodactylum tricornutum
Entamoeba histolytica
Leishmania major
Cryptosporidium parvum
Ostreococcus lucimarinus
Nannochloropsis gaditana
Komagataella pastoris
Naumovozyma dairenensis
Saccharomyces cerevisiae
DUSEL4 Archaeon
0.2

Rps20p

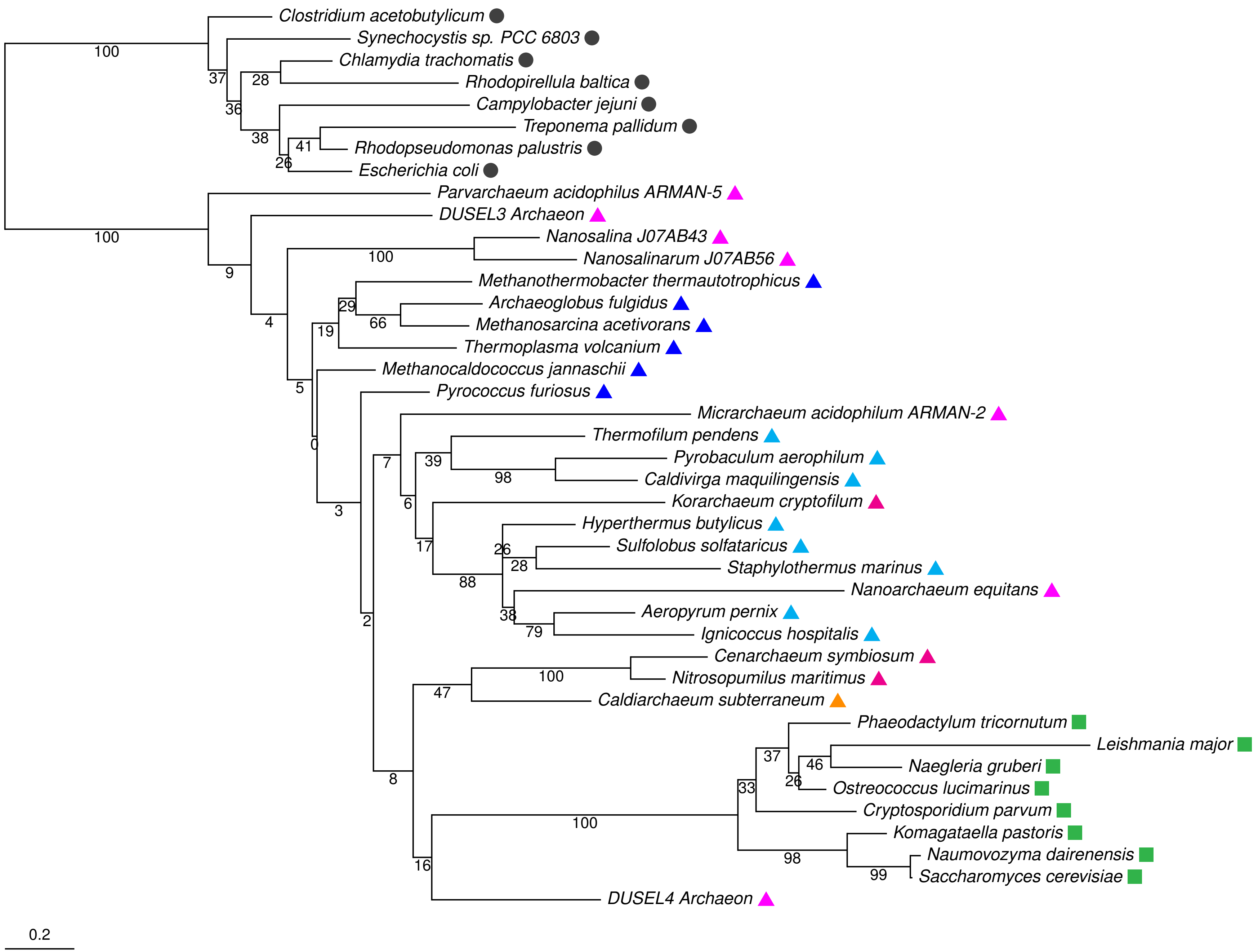
Clostridium acetobutylicum
Synechocystis sp. PCC 6803
Chlamydia trachomatis
Rhodopirellula baltica
Campylobacter jejuni
Treponema pallidum
Rhodopseudomonas palustris
Escherichia coli
Parvarchaeum acidophilus ARMAN-5
DUSEL3 Archaeon
Nanosalina J07AB43
Nanosalinarum J07AB56
Methanothermobacter thermautotrophicus
Archaeoglobus fulgidus
Methanosarcina acetivorans
Thermoplasma volcanium
Methanocaldococcus jannaschii
Pyrococcus furiosus
Micrarchaeum acidophilum ARMAN-2
Thermofilum pendens
Pyrobaculum aerophilum
Caldivirga maquilingensis
Korarchaeum cryptofilum
Hyperthermus butylicus
Sulfolobus solfataricus
Staphylothermus marinus
Nanoarchaeum equitans
Aeropyrum pernix
Ignicoccus hospitalis
Cenarchaeum symbiosum
Nitrosopumilus maritimus
Caldiarchaeum subterraneum
Phaeodactylum tricornutum
Leishmania major
Naegleria gruberi
Ostreococcus lucimarinus
Cryptosporidium parvum
Komagataella pastoris
Naumovozyma dairenensis
Saccharomyces cerevisiae
DUSEL4 Archaeon
100
37
28
36
38
41
26
100
9
100
4
29
19
66
5
0
3
7
39
98
6
17
26
28
88
38
79
2
100
47
37
46
26
33
100
98
99
8
16
0.2

Bacterial genes

Guf1p

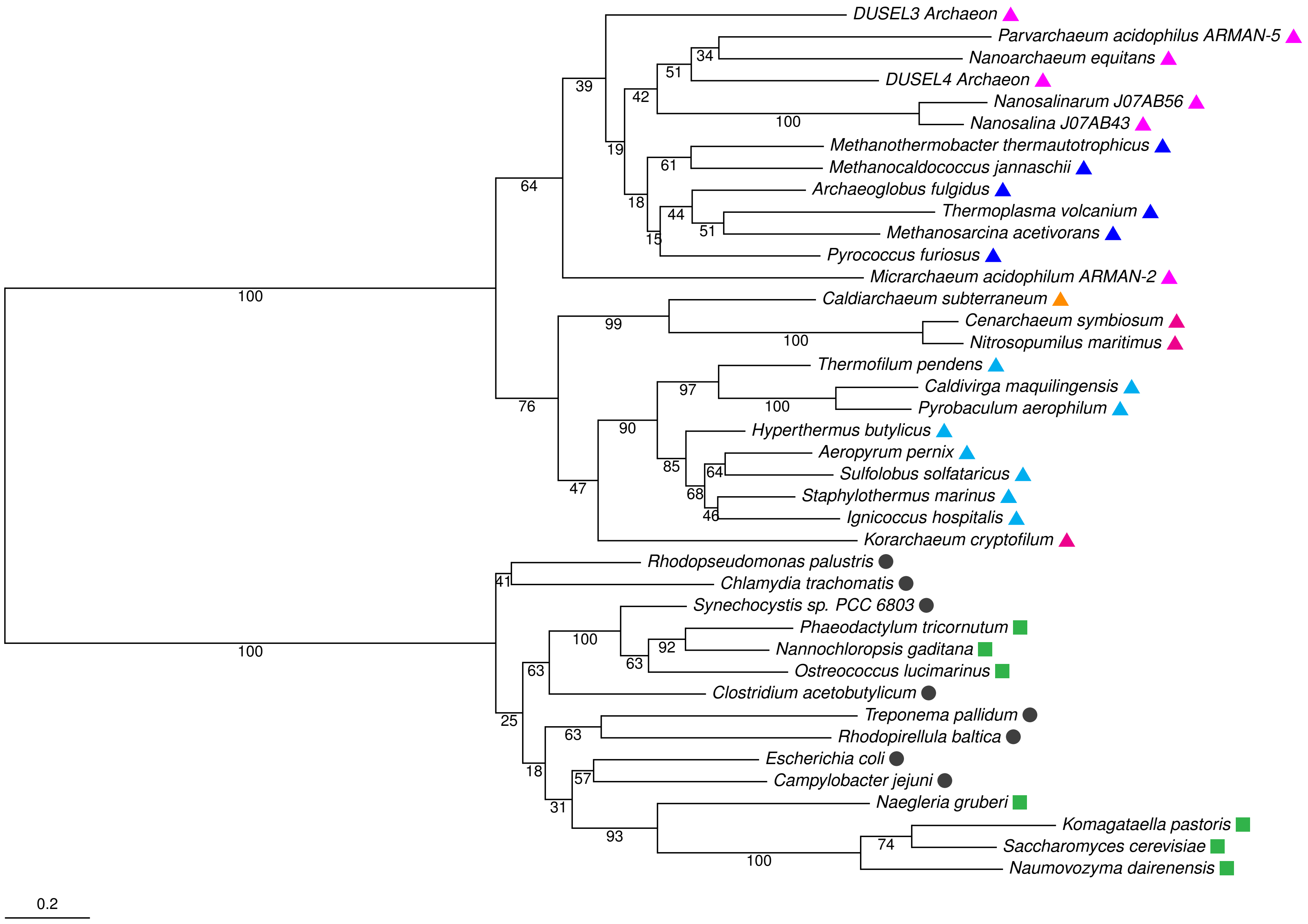

Tpi1p

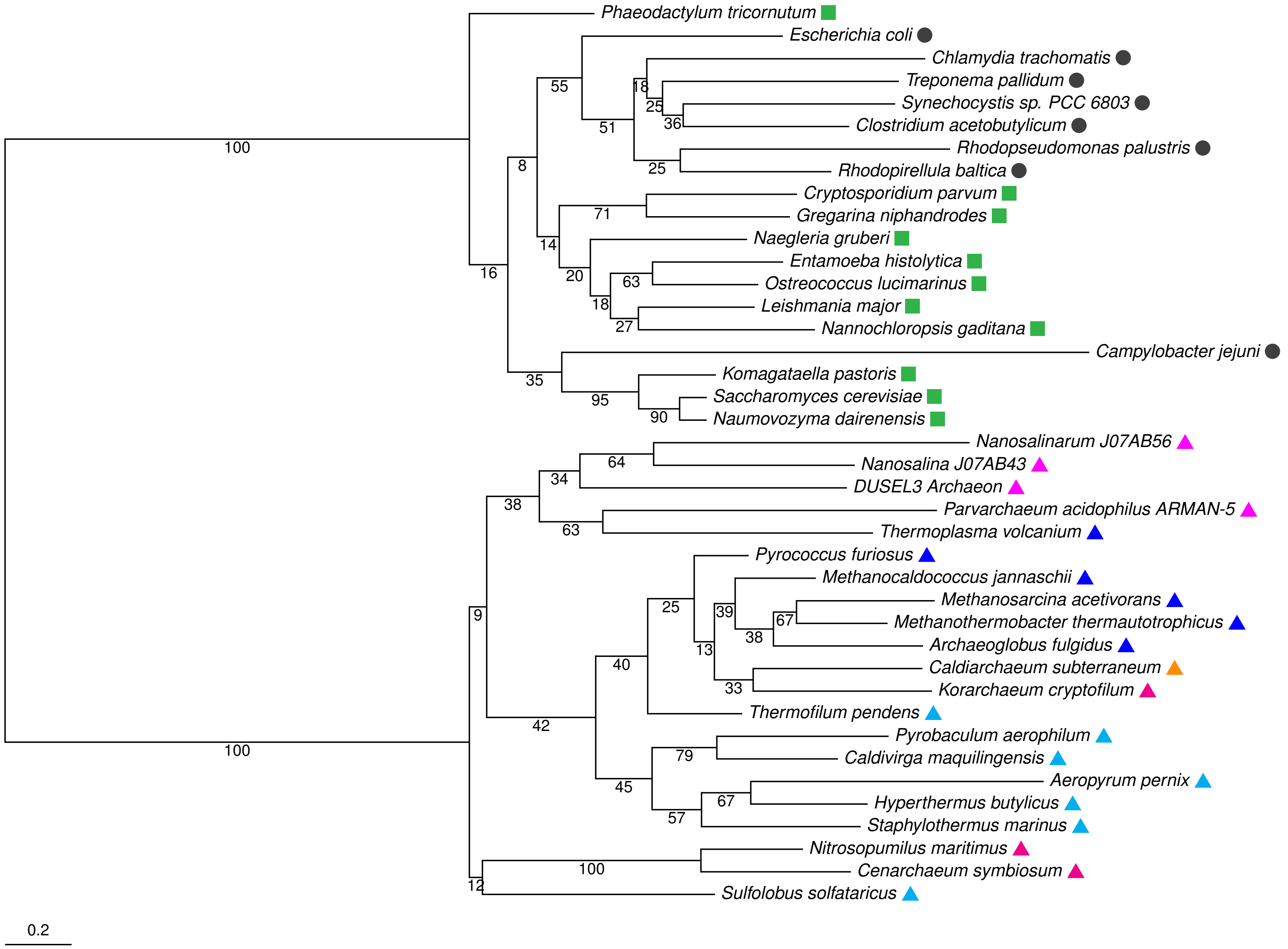

Phaeodactylum tricornutum
Escherichia coli
Chlamydia trachomatis
Treponema pallidum
Synechocystis sp. PCC 6803
Clostridium acetobutylicum
Rhodopseudomonas palustris
Rhodopirellula baltica
Cryptosporidium parvum
Gregarina niphandrodes
Naegleria gruberi
Entamoeba histolytica
Ostreococcus lucimarinus
Leishmania major
Nannochloropsis gaditana
Campylobacter jejuni
Komagataella pastoris
Saccharomyces cerevisiae
Naumovozyma dairenensis
Nanosalinarum J07AB56
Nanosalina J07AB43
DUSEL3 Archaeon
Parvarchaeum acidophilus ARMAN-5
Thermoplasma volcanium
Pyrococcus furiosus
Methanocaldococcus jannaschii
Methanosarcina acetivorans
Methanothermobacter thermautotrophicus
Archaeoglobus fulgidus
Caldiarchaeum subterraneum
Korarchaeum cryptofilum
Thermofilum pendens
Pyrobaculum aerophilum
Caldivirga maquilingensis
Aeropyrum pernix
Hyperthermus butylicus
Staphylothermus marinus
Nitrosopumilus maritimus
Cenarchaeum symbiosum
Sulfolobus solfataricus
0.2

Non-conserved genes

Rps23bp

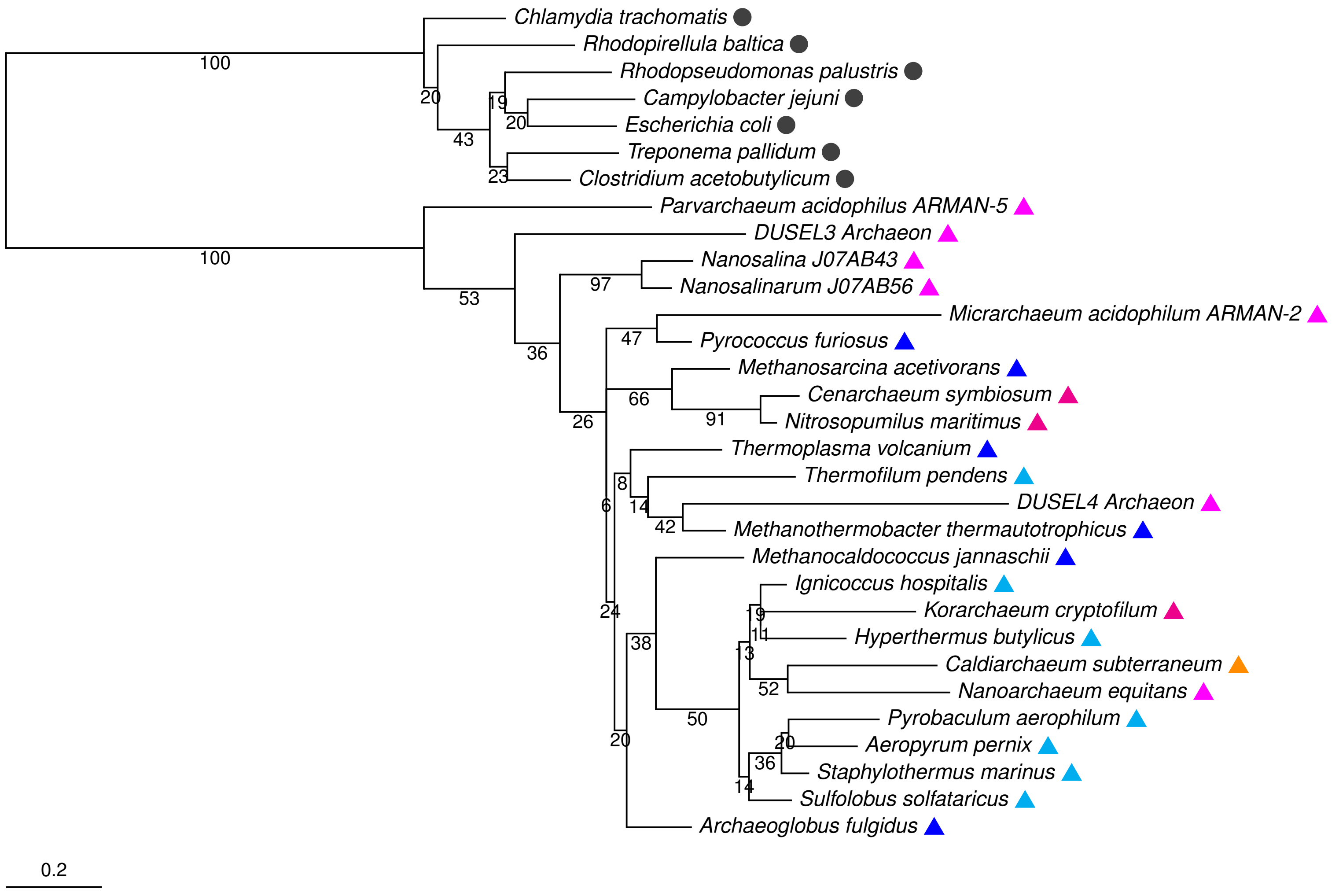

E. coli dITP/XTP pyrophosphatase

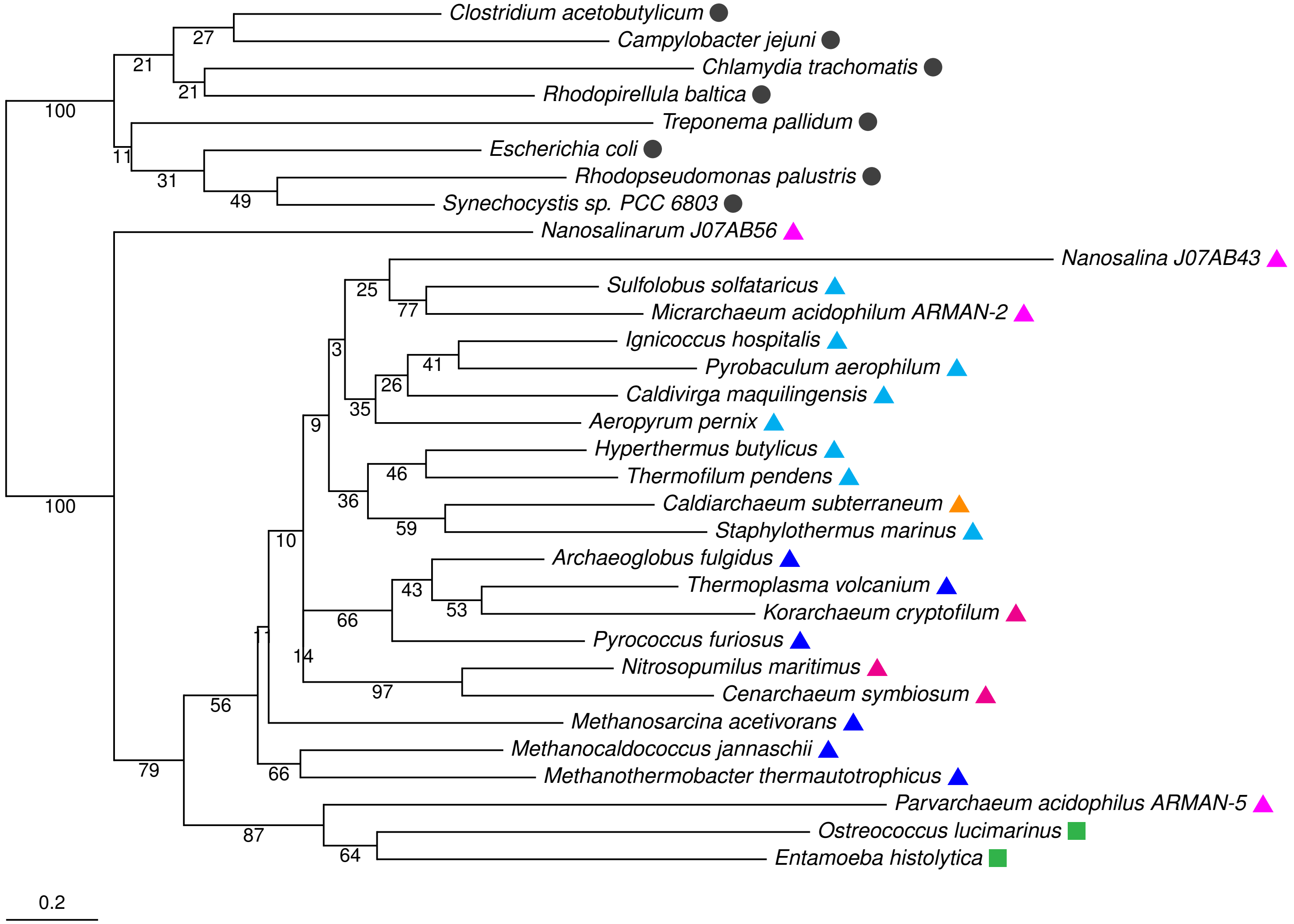

Frs1p

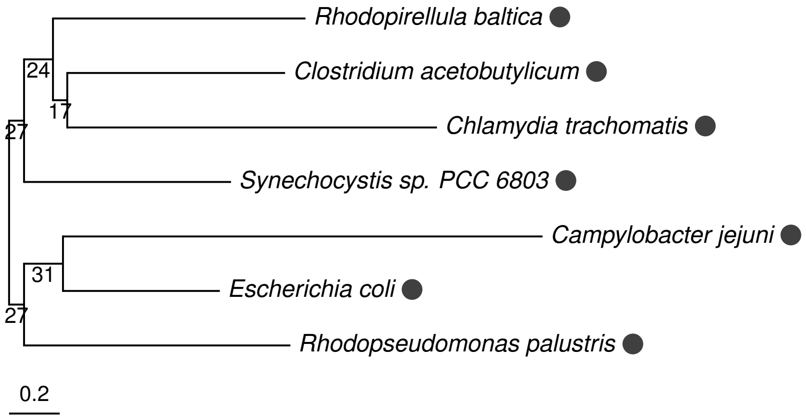
Rhodopirellula baltica
Clostridium acetobutylicum
Chlamydia trachomatis
Synechocystis sp. PCC 6803
Campylobacter jejuni
Escherichia coli
Rhodopseudomonas palustris
24
17
27
31
27
0.2

Rpl5p

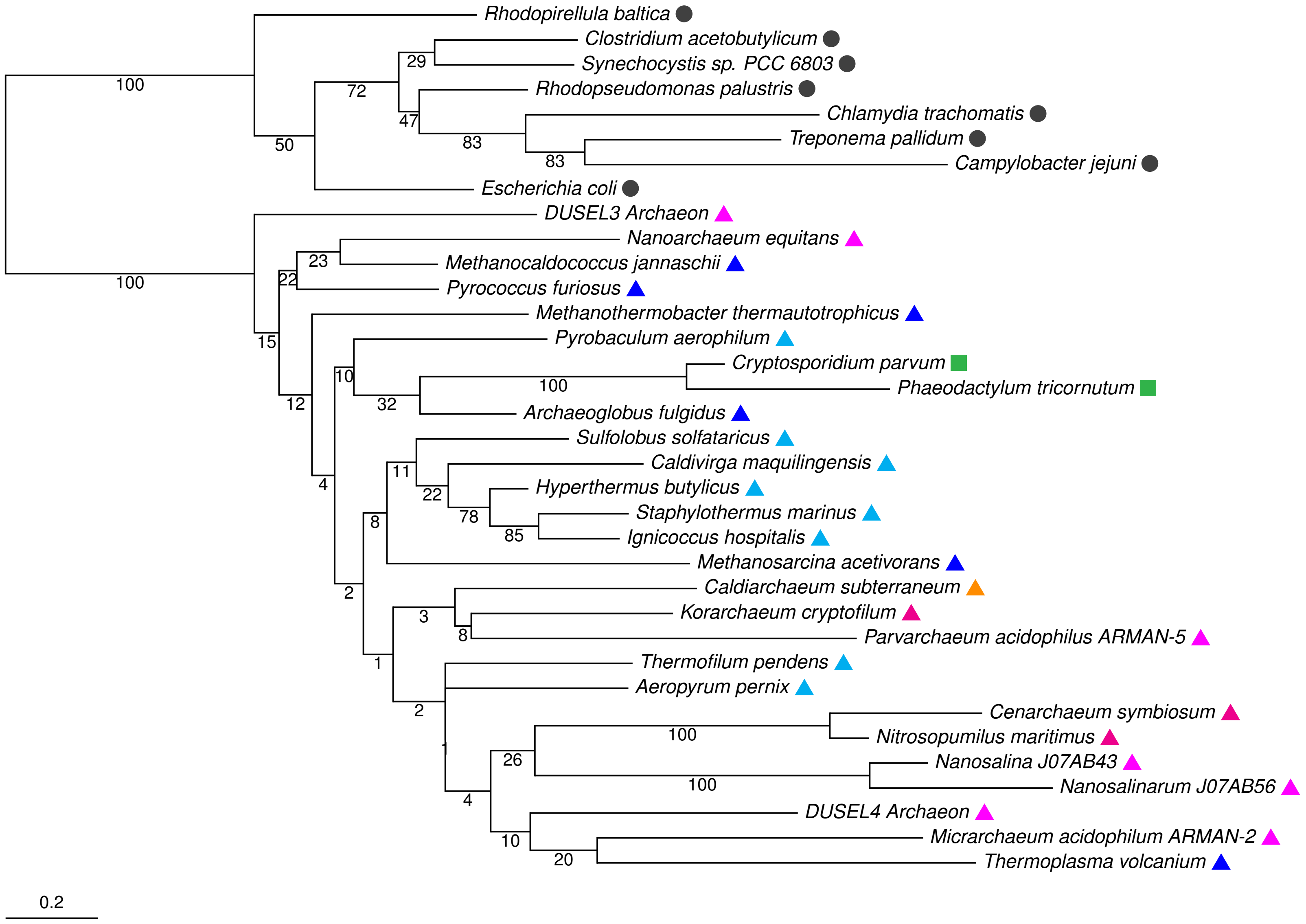

Rhodopirellula baltica
Clostridium acetobutylicum
Synechocystis sp. PCC 6803
Rhodopseudomonas palustris
Chlamydia trachomatis
Treponema pallidum
Campylobacter jejuni
Escherichia coli
DUSEL3 Archaeon
Nanoarchaeum equitans
Methanocaldococcus jannaschii
Pyrococcus furiosus
Methanothermobacter thermautotrophicus
Pyrobaculum aerophilum
Cryptosporidium parvum
Phaeodactylum tricornutum
Archaeoglobus fulgidus
Sulfolobus solfataricus
Caldivirga maquilingensis
Hyperthermus butylicus
Staphylothermus marinus
Ignicoccus hospitalis
Methanosarcina acetivorans
Caldiarchaeum subterraneum
Korarchaeum cryptofilum
Parvarchaeum acidophilus ARMAN-5
Thermofilum pendens
Aeropyrum pernix
Cenarchaeum symbiosum
Nitrosopumilus maritimus
Nanosalina J07AB43
Nanosalinarum J07AB56
DUSEL4 Archaeon
Micrarchaeum acidophilum ARMAN-2
Thermoplasma volcanium
100
100
50
72
29
47
83
83
23
22
15
12
10
32
100
4
11
22
78
85
8
2
3
8
1
2
100
100
26
4
10
20
0.2
