# Supplementary material for: Archaeal “Dark Matter” and the Origin of Eukaryotes
Source: Genome Biol Evol. 2014 Feb 14;6(3):474–81. doi: 10.1093/gbe/evu031 (PMC3971582; doi:10.1093/gbe/evu031)
Supplement: Supplementary Data [file supp_evu031_williams_etal_suppl.docx]

**Archaeal “dark matter” and the origin of eukaryotes**

Tom A. Williams and T. Martin Embley

**Supplementary Tables**

In the following tables, as well as in the accompanying single gene tree diagrams, the yeast gene names (e.g. Rpl3p) are used as a common reference point.

**Supplementary Table 1: Mitochondrial and plastid contamination in the single gene phylogenies inferred from the Rinke et al. 38-gene concatenation.** “Archaeal monophyly” refers to whether the archaeal sequences form a clan in the unrooted tree, in agreement with the three-domains hypothesis. Yeast gene names are included in parentheses.

| **Gene** | **Eukaryotes represented by a bacterial (mitochondrial or plastid) copy** | **Archaeal monophyly (Yes/No/Not applicable)** |
| --- | --- | --- |
| 60S L23A (Rpl23bp) | *Naumovozyma* | No |
| Kae1 (Kae1p) | *Naumovozyma*, *Komagataella* | No |
| 40S S3 (Rps3p) |  | No |
| 40S S2 (Rps2p) | *Phaeodactylum* | No |
| 60S L9 (Rpl9p) | *Ostreococcus* | No |
| 60S L1a (Rpl1ap) |  | Yes |
| 40S S15 (Rps15p) |  | No |
| 60S L17-like (Rpl17ap) | *Naumovozyma, Nannochloropsis* | No |
| 37S Mrp4 (Mrps9p) | All eukaryotes | N/A |
| 54S Yml19 (Rpl12ap) | *Naumovozyma*, *Saccharomyces*, *Komagataella, Phaeodactylum* | Yes |
| Srp54 (Srp54p) |  | No |
| 60S L4a (Rpl4bp) |  | Yes |
| L37 Mrps17 (Rps11ap) | *Ostreococcus, Saccharomyces* | No |
| Ura8 (Ura8p) |  | No |
| 54S Rml2 (Rpl2bp) | *Saccharomyces* | No |
| 37S Mrps28 (Mrps28p) | All eukaryotes | N/A |
| 40S S16 (Rps16ap) | *Naegleria, Ostreococcus* | No |
| L18 L5 (Rpl5p) | (Only two eukaryotes in tree) | No |
| 54S Yml5/Yml7 (Rpl11ap) | All eukaryotes | N/A |
| Rsm7 (Rps5p) | *Saccharomyces* | No |
| Guf1 (Guf1p) | All eukaryotes | Yes |
| Fun12 (Fun12p) | *Phaeodactylum, Komagataella, Naumovozyma, Ostreococcus* | No |
| 60S L26a (Rpl26bp) | *Ostreococcus, Nannochloropsis, Phaeodactylum, Naegleria* | N/A |
| 40S S14a (Rps14bp) |  | No |
| 50S L10 (-) | *Phaeodactylum* | N/A |
| 54S Yml47 (Rpl10p) | All eukaryotes | N/A |
| 37S Mrps8 (Mrps8p) | All eukaryotes | N/A |
| 54S Yml9 (Rpl3p) | *Nannochloropsis, Ostreococcus, Naegleria, Komagataella, Saccharomyces* | No |
| 40S 18a (Rps18bp) | *Ostreococcus* | No |
| Phenyl-tRNA ligase (Frs2p) | *Phaeodactylum, Ostreococcus, Nannochloropsis* | No |
| 54S Yml10/18 (Mrpl10p) | *Nannochloropsis, Naumovozyma, Saccharomyces, Phaeodactylum, Leishmania* | No |
| 40S S20 (Rps20p) |  | No |
| 30S S12 (Rps23bp) | (No eukaryotes in tree) | N/A |
| TPI (Tpi1p) | (This is a bacterial gene, archaeal copy distantly related) | Yes |
| Ribosyltransferase (Mrpl10p) | All eukaryotes | Yes |
| Pyrophosphatase (Rps23bp) | (Only two eukaryotes in tree) | No |
| Escherichia coli Queuine tRNA-ribosyltransferase | All eukaryotes | Yes |
| Rnh201 (Rnh201p) | *Ostreococcus, Gregarina* | No |
| Phenyl-tRNA synthetase (Frs1p) | (No eukaryotes or Archaea in tree) | N/A |

**Supplementary Table 2: *Saccharomyces cerevisiae* mitochondrial genes included in the 38-gene concatenation of Rinke et al.** The gene name of the orthologous nucleo-cytosolic *S. cerevisiae* sequence is provided in parentheses.

| **Gene annotation** | **Position in original concatenation** |
| --- | --- |
| mitochondrial 37S ribosomal protein MRP4 (Rps0ap) | 1083-1296 |
| mitochondrial 54S ribosomal protein YmL19 (-) | 1297-1427 |
| mitochondrial 37S ribosomal protein MRPS17 (-) | 1972-2042 |
| mitochondrial 54S ribosomal protein RML2 (Rpl2bp) | 2561-2758 |
| mitochondrial 37S ribosomal protein MRPS28 (-) | 2759-2827 |
| mitochondrial 54S ribosomal protein YmL7/YmL5 (-) | 2939-3086 |
| Rsm7p (Rps5p) | 3087-3219 |
| Guf1 (-) | 3220-3810 |
| mitochondrial 54S ribosomal protein YmL47 (Rpl10p) | 4447-4570 |
| mitochondrial 37S ribosomal protein MRPS8 (-) | 4571-4688 |
| mitochondrial 54S ribosomal protein YmL9 (Rpl3p) | 4689-4888 |
| mitochondrial 54S ribosomal protein YmL10/YmL18 (-) | 5196-5325 |

**Supplementary Table 3: Overlap in the composition of the Williams, et al. 2012 dataset (the 29-gene dataset we update here with the new archaeal lineages), the 38 genes used in the original concatenation of Rinke, et al. 2013, and the 20-gene version of that dataset from which contaminants had been replaced or removed.** The numbers in brackets correspond to the (arbitrary) gene numbers used in the two analyses, which is the same as the numbering of the alignments and single gene trees available for download at DataDryad. Italics denote cases in which a gene was included in one dataset but not the other. Rinke, et al. genes that were included in the revised, 20-gene contamination-free dataset are indicated with an asterisk.

| **Gene (*S. cerevisiae* gene ID unless otherwise indicated)** | Williams, et al. 2012 | Rinke, et al. 2013 |
| --- | --- | --- |
| Rps14bp | Y (1) | Y (24)* |
| Rps23bp | Y (6) | Y (33)* |
| Fun12p | Y (14) | Y (22) |
| Rpl11ap | Y (15) | Y (19)* |
| Rps3p | Y (20) | Y (3)* |
| Rps16ap | Y (22) | Y (17)* |
| Rpl1ap | Y (24) | Y (6)* |
| Rpl2bp | Y (29) | Y (15) |
| Rpl23bp | Y (30) | Y (1) |
| Rpl12ap | Y (31) | Y (10) |
| Eft1p | Y (33) | Y (21); distant paralogue |
| Kae1p | Y (34) | Y (2)* |
| Rps0bp | Y (35) | Y (9) |
| Rps5p | Y (36) | Y (20)* |
| Rps2p | Y (37) | Y (4)* |
| Srp54p | Y (40) | Y (11)* |
| *Tef1p* | *Y (4)* | *N* |
| *Rli1p* | *Y (5)* | *N* |
| *Dps1p* | *Y (10)* | *N* |
| *Rpa190p* | *Y (11)* | *N* |
| *Sec61p* | *Y (12)* | *N* |
| *Cct5p* | *Y (16)* | *N* |
| *Rfc2p* | *Y (17)* | *N* |
| *Vma2p* | *Y (23)* | *N* |
| *Map2p* | *Y (25)* | *N* |
| *Rpl16ap* | *Y (28)* | *N* |
| *Gln4p* | *Y (32)* | *N* |
| *Rpa135p* | *Y (39)* | *N* |
| *Srp101p* | *Y (41)* | *N* |
| *Rpl9p* | *N* | *Y (5)** |
| *Rps15p* | *N* | *Y (7)** |
| *Rpl17ap* | *N* | *Y (8)* |
| *Rpl4bp* | *N* | *Y (12)* |
| *Rps11ap* | *N* | *Y (13)** |
| *Ura8p* | *N* | *Y (14)** |
| *Mrps28p* | *N* | *Y (16)** |
| *Rpl5p* | *N* | *Y (18)** |
| *Rpl26b* | *N* | *Y (23)* |
| *Escherichia coli ribosomal protein L10* | *N* | *Y (25)* |
| *Rpl10p* | *N* | *Y (26)* |
| *Mrps8p* | *N* | *Y (27)** |
| *Rpl3p* | *N* | *Y (28)* |
| *Rps18bp* | *N* | *Y (29)* |
| *Frs2p* | *N* | *Y (30)** |
| *Mrpl10p* | *N* | *Y (31)* |
| *Rps20p* | *N* | *Y (32)** |
| *Tpi1p* | *N* | *Y (34)* |
| *Escherichia coli Queuine tRNA-ribosyltransferase* | *N* | *Y (35)* |
| *Escherichia coli dITP/XTP pyrophosphatase* | *N* | *Y (36)** |
| *Rnh201p* | *N* | *Y (37)* |
| *Frs1p* | *N* | *Y (38)* |
